# Formation and Coherent Propagation of Femtosecond-Laser-Induced Periodic Surface Structures (LIPSS) in Fluorine-Doped Tin Oxide: Control, Potential Applications, and Challenges

**DOI:** 10.1021/acsami.6c06535

**Published:** 2026-06-22

**Authors:** Gonzalo Gomez-Munoz, Rocio Ariza, Fernando Nuñez-Galvez, Victor López-Flores, Fatima Cabello-Pardos, Belen Sotillo, Carlos Prieto, Jose Gonzalo, Francisco Javier Garcia-Lopez, Paloma Fernández Sánchez, Carmen Lopez-Santos, Javier Solis

**Affiliations:** † 16379 Instituto de Óptica-CSIC (IO−CSIC), c/Serrano 121, Madrid ES-28006, Spain; ‡ 16778 Instituto de Ciencia de Materiales de Sevilla-US-CSIC (ICMSE-US-CSIC), C/Américo Vespucio 49, Seville ES-41092, Spain; § Departamento de Física Aplicada I-Escuela Politécnica Superior, Universidad de Sevilla, c/Virgen de África, Seville ES-41013, Spain; ∥ Departamento de Física de Materiales, UCM, Pl. de las Ciencias 1, Madrid ES-28040, Spain; ⊥ Instituto de Ciencia de Materiales de Madrid-CSIC (ICMM-CSIC), Cantoblanco, Madrid ES-28049, Spain; # Centro Nacional de Aceleradores, Av. Tomas. Edison 7, Seville ES-41092, Spain; ∇ Departamento de Física Atómica Molecular y Nuclear, Universidad de Sevilla, Seville ES-41012, Spain

**Keywords:** FTO, TCOs, LIPSS, electrical anisotropy, fs-laser processing, large area devices, thermoelectric devices

## Abstract

We have analyzed the formation and coherent propagation of laser-induced periodic surface structures (LIPSS) upon fs-laser irradiation of fluorine-doped tin oxide (FTO) films. The aim is the generation of large electrical anisotropies in macroscopic areas for applications including laser-written transparent heaters, sensors, or substrates for electrically controllable wettability, among others. The films have been processed with high-repetition-rate (hundreds of kHz) fs-laser pulses at 1030 nm using different laser (fluence and pulse duration) and scanning parameters (speed and line overlap). Optimal LIPSS formation and coherent propagation are conditioned by the dynamic evolution of the F content in the laser-processed regions that can be controlled via processing parameters. However, the homogeneity in the spatial distribution of F in the microscale in the pristine samples can generate important issues that are carefully considered and discussed in the present work. Still, the formation of optically and electrically highly anisotropic surfaces for laser scanning speeds well above 1.0 m/s has been demonstrated, reaching electrical resistivity anisotropy factors of >10^3^. Consequently, the potential development of large-area applications using LIPSS-structured FTO films seems feasible, as we have demonstrated by fabricating an efficient laser-written electrothermal transparent device as a proof-of-concept.

## Graphical abstract

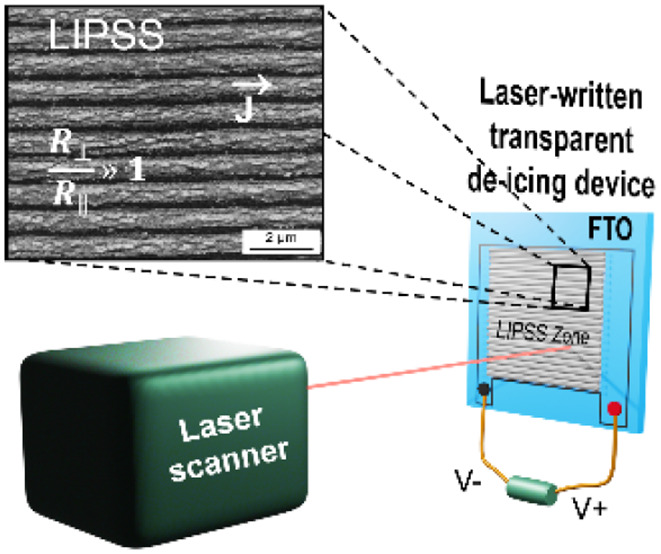

## 1 Introduction

Transparent conductive oxides (TCOs) are materials with simultaneous electrical conductivity at room temperature (RT) and low optical absorptance in the visible region,^1^ which makes them essential for applications involving charge transfer and light management like OLED’s,^2^ flat-panel displays,^3^ energy harvesting and photovoltaics,^4,5^ smart windows, and transparent heaters.^6^ TCOs are typically oxide semiconductors with a bandgap above 3 eV. This property confers them transparency in the visible—VIS—and infrared—IR—regions of the spectrum and, when conveniently doped or alloyed, allows inducing shallow electronic levels in the bandgap, generating relatively high carrier’s concentration at RT (typically ≥10^20^ cm^–3^). These features enable them to exhibit simultaneously VIS-NIR transparency and low resistivity (∼10^–3^–10^–4^ Ω·cm).

Among the different TCOs presently available at the commercial level, F-doped SnO_2_ (FTO) appears as an excellent alternative in applications requiring a performance not as demanding as that of ITO,^1^ AZO,^7^ or GZO.^8^ An inherent self-compensation mechanism limits the free electron density, mobility, and conductivity values achievable in FTO.^9^ As a consequence, ITO has a lower sheet resistance and higher room-temperature electron density than FTO. However, FTO has a relatively low cost, high chemical resistance, excellent thermal stability, and a good combination of high transparency and low resistivity. All of which confer it a prominent role in optoelectronics, energy harvesting, and energy storage devices.^10^

On the other hand, the production of electrically anisotropic TCO surfaces (i.e., optically transparent surfaces with alternating highly conductive and highly resistive or insulating regions) may facilitate the production of large area transparent conductive flexible paths,^11^ transparent heaters,^3^ photothermal energy conversion^12^ and management devices,^13^ sensors,^14^ large area templates for the production of aligned metal nanostructures by electrochemical deposition,^15^ substrates for electrically controllable wettability,^16^ or the development of fluorescence anisotropies in perovskite films.^17^ In this respect, fs-laser processing offers rapid prototyping and low-cost methodologies for surface structuring of TCOs, and it has been used for a variety of applications. They include the production of strong optical form birefringence in ITO^18^ and FTO^19^ films or the use of nanostructured ITO for liquid crystal alignment and the development of high-transparency electrodes.^20,21^ Similarly, fs-laser annealing has been used to improve the performance of FTO in perovskite solar cells,^22^ and direct laser interference patterning (DLIP) using short and ultrashort laser pulses has been used to improve the optical performance of FTO^21,23^ and ITO,^24^ in this latter case as a front electrode in solar cells.

None of these works are focused on the development of electrical anisotropies as a consequence of the formation of laser-induced periodic surface structures (LIPSS).^25^ The formation of LIPSS using ultrafast lasers^26,27^ enables the formation of macroscopic areas with a microscopic periodic variation of topography, structure, and/or composition, and thus the development of different types of surfaces with anisotropic properties.^28^ Only recently, we have demonstrated the formation and coherent propagation of LIPSS in ITO films and analyzed in detail the role of the compositional changes (mainly preferential In loss) in the evolution of the LIPSS as laser pulses accumulate.^29^

It must be emphasized that LIPSS formation is a self-organization, universal phenomenon^25^ that has been observed in every kind of material, including metals^30,31^ (very specially in hard-to-process ones like W and Zr^32,33^ ), semiconductors,^34,35^ dielectrics, or polymers.^36^ Their formation mechanisms for different materials and laser processing conditions have been analyzed by many authors^37–39^ and remain controversial in several cases. Still, LIPSS’s intrinsic characteristics (self-organization, scalability, low energy consumption) have made it possible to develop a number of applications in photovoltaics,^40^ surface wetting control,^41^ sensing devices,^42^ tribological properties,^43^ and, very specially, in the frame of biomimetics.^44^

In this work, we have analyzed the formation and coherent propagation of LIPSS upon fs-laser irradiation of commercial FTO films (NSG TEC-15) aiming at producing highly anisotropic resistivity surfaces. The role of sample preparation and laser processing parameters in the morphology, structural, compositional, optical, and electrical properties of the processed surfaces has been analyzed, with special emphasis on the role of the F-content evolution in the processed regions and the induced electrical anisotropies. As a consequence of the optimization process, we have generated very large electrical conduction anisotropies of use in large-area devices, as demonstrated in a proof-of-concept transparent electrothermal deicing prototype laser-written in FTO. The challenges of this approach for the production of devices in FTO films based on the coherent propagation of LIPSS, and the fine control of the properties of the so-generated structures are analyzed in detail.

## 2 Results and Discussion

### 2.1 Determination of the Laser Processing Window

Laser processing was used to texture the FTO film surfaces in square regions of various sizes, varying the laser repetition rate (*f*_rep_), pulse duration (τ), laser fluence (*F*), scan line separation (*d*), and scanning speed (*v*). Figure 1 includes an illustrative example of a low-magnification Scanning Electron Microscopy (SEM) micrograph showing the formation and coherent propagation of LIPSS in a TEC-15 sample upon high-repetition rate fs-laser irradiation at 1030 nm. The coherent propagation of LIPSS can be induced for a relatively broad range of processing conditions with pulses ranging from 290 fs up to 420 fs (maximum interval analyzed) when the laser polarization is perpendicular to the beam scanning direction,^45,46^ as shown in Figure 1. Large-scale homogeneously LIPSS-covered surfaces can be produced, leading to a characteristic diffraction-colored appearance (see the inset image of Figure 1). As indicated, LIPSS are propagated continuously over macroscopic lengths (several cm) at scanning speeds well above 1 m/s (at least up to 4 m/s) as we observed previously in ITO,^29^ a rather different TCO in terms of composition since SnO_2_ is a minority component of ITO, with a content typically around 10 wt %.

**Figure 1:**
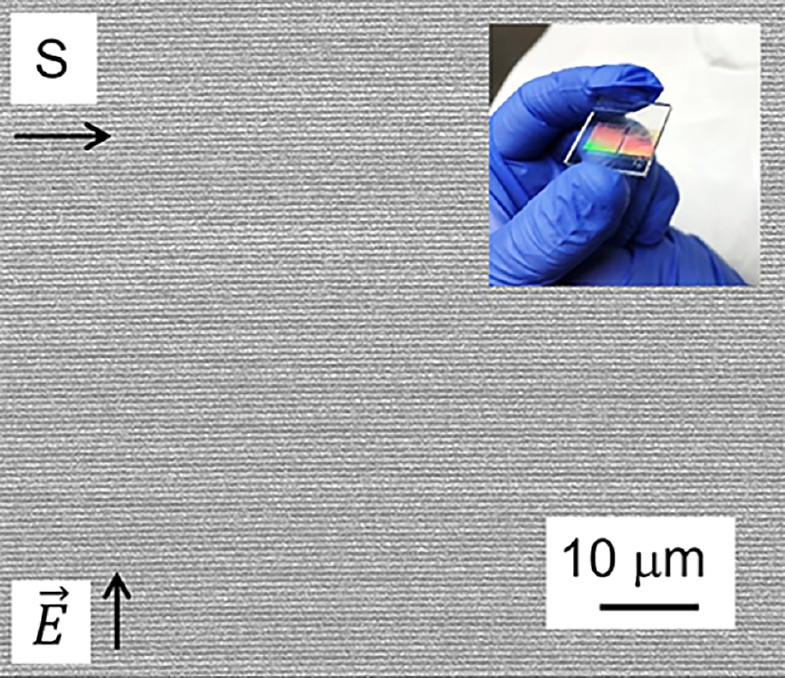
Low magnification SEM image of a TEC-15 film processed with an fs-laser (*f*_rep_ = 500 kHz, τ = 290 fs, *d* = 4 μm, *v* = 1.9 m/s, *F* = 369 mJ/cm^2^). The arrows indicate the polarization of the laser beam (*E⃗*) and the scan direction (S). The inset image shows a picture of the sample taken with a color camera.

Figure 2 shows the characteristic morphology of the irradiated regions for a laser repetition rate *f*_rep_ = 500 kHz for the three different pulse durations studied (290 fs, 350 fs, and 420 fs), and a low fluence above but close to the modification threshold (all the images correspond to *d* = 4 μm and *v* = 2.5 m/s). SEM observations indicate that that the minimum fluence for LIPSS formation is *F*_LIPSS‑Min_ ≈ 525 mJ/cm^2^. This value, for the indicated values of *f*_rep_, *d*, and *v*, is independent of the pulse duration in the studied interval, something likely related to the small linear absorption of the material at 1030 nm due to free carrier absorption (FCA) at room temperature. Atmospheric Pressure Chemical Vapor Deposition (APCVD) and spray pyrolysis-deposited FTO films on heated substrates show carrier densities at room temperature in the range of 10^20^ cm^–3^,^47^ with skin depth values typically around *k* ≈ 0.05 at 1030 nm. This small energy absorption seems to trigger a subsequent avalanche ionization process and the early development of quasi-periodic structures perpendicular to the laser field. However, in these conditions poor propagation is observed.

**Figure 2:**
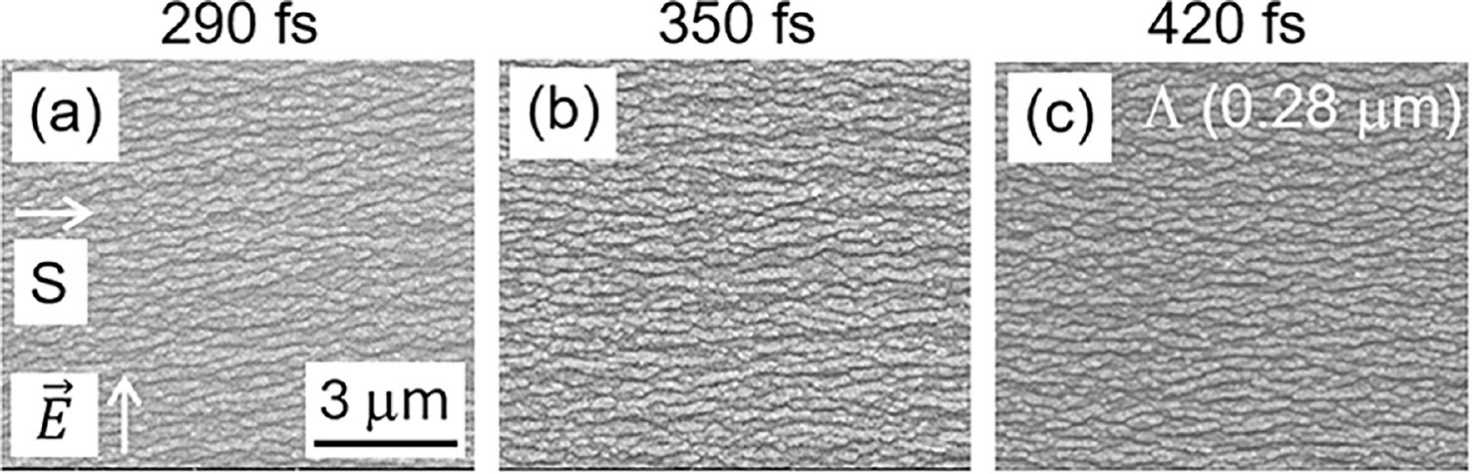
((a)–(c)) SEM images at the same magnification of TEC-15 samples processed with 500 kHz, fs-laser pulses at 525 mJ/cm^2^ for the indicated pulse durations: 290 fs (a), 350 fs (b), and 420 fs (c). The arrows indicate the orientations of the polarization of the laser (E⃗) and the beam scan direction (S). The FFT-measured period of the structures (Λ) is the same in all cases.

The initial modification mechanism, associated with the combination of low linear absorption followed by avalanche, seems to be supported by the transformation thresholds observed by other authors in laser-processed FTO films at different pulse durations and repetition rates. Longer pulse durations, i.e., in the ps-range, substantially increase the modification threshold^21^ above 1 J/cm^2^ at 1064 nm (10 kHz rep. rate), while subpicosecond pulses at high-repetition rate (100 kHz) yield a much lower modification threshold at 1030 nm,^48^ which is consistent to the one we have observed. Irradiation of FTO with low repetition rate (5 kHz) fs-laser pulses at 514 nm showed though a linear dependence of the fluence ablation threshold with the pulse duration in the 280 fs-1.0 ps^49^ range, which seems to contradict our observation of a nearly constant LIPSS-formation threshold fluence in the 350–420 fs pulse duration interval. This discrepancy is likely related to two main factors. On the one hand, there is an enormous difference in laser repetition rates involved in both experiments (5 kHz vs 500 kHz). On the other hand, FTO is transparent at 515 nm, which requires multiphoton ionization to couple the laser energy in the surface (the ablation threshold at 514 nm is ∼0.84 J/cm^2^) while the material shows free carrier absorption at 1030 nm that favors a subsequent avalanche process and a much lower LIPSS-formation threshold in our case (0.52 J/cm^2^). This initial modification mechanism (FCA followed by avalanche) is also consistent to previous observations in fs-laser processed ITO-films^29^ although the subsequent LIPSS formation and evolution are obviously conditioned by the different composition of both materials.

At these low fluences, the early development of LIPSS having a characteristic period (Λ_S_) close to 0.3 μm can be observed. Given the orientation of the structures with respect to the laser beam polarization, these might be considered as Low Spatial Frequency (LSF) LIPSS; however, their period is much smaller than the laser wavelength, which makes it difficult to consider them as either High or Low Spatial Frequency LIPSS, as discussed by Heffner et al.^48^ Still, the observed period seems to be independent of the pulse duration used within the studied interval. As soon as the fluence is increased the induced structures show clear differences with respect to those induced just above the LIPSS formation threshold.

Figure 3 includes SEM micrographs at the same intermediate magnification of TEC-15 films irradiated at *f*_rep_ = 500 kHz with laser fluences in the 560–650 mJ/cm^2^ for the three pulse durations analyzed. For fluences above 520 mJ/cm^2^, the previously described structure having a period Λ_S_ of ∼0.3 μm evolves showing regularly spaced valleys with a characteristic spatial frequency slightly above 0.8 μm (Λ_L_ ∼ 0.83 μm) in all cases. Schematically, we observe that the formation of LIPSS with well-defined valleys occurs for lower fluences as the pulse duration is shorter (cf. images for 290 and 420 fs pulses at 590 mJ/cm^2^). This suggests that the development of LSF-LIPSS with “long period” (Λ_L_ ∼ 0.83 μm) is conditioned both by the extent of the avalanche effects induced and by the maximum transient peak temperature achieved in each case. If, as above indicated, the material modification occurs via FCA followed by avalanche, stronger avalanche effects are expected for the shortest pulse duration, while the morphology changes should be smoother as the pulse duration increases. At the same time, the stronger avalanche effects also lead to “splitting” and breakage of the LSF-LIPSS at 620 mJ/cm^2^ while for this fluence, at 420 fs, clearly marked valleys are observed for the first time. The same applies for the observation of strong ablation effects that are clear at 650 mJ/cm^2^ for the shortest pulse duration and only start to appear for this fluence at 420 fs. Interestingly, the evolution of Λ_S_ and Λ_L_ with fluence does not show appreciable changes (cf. Figure 3).

**Figure 3:**
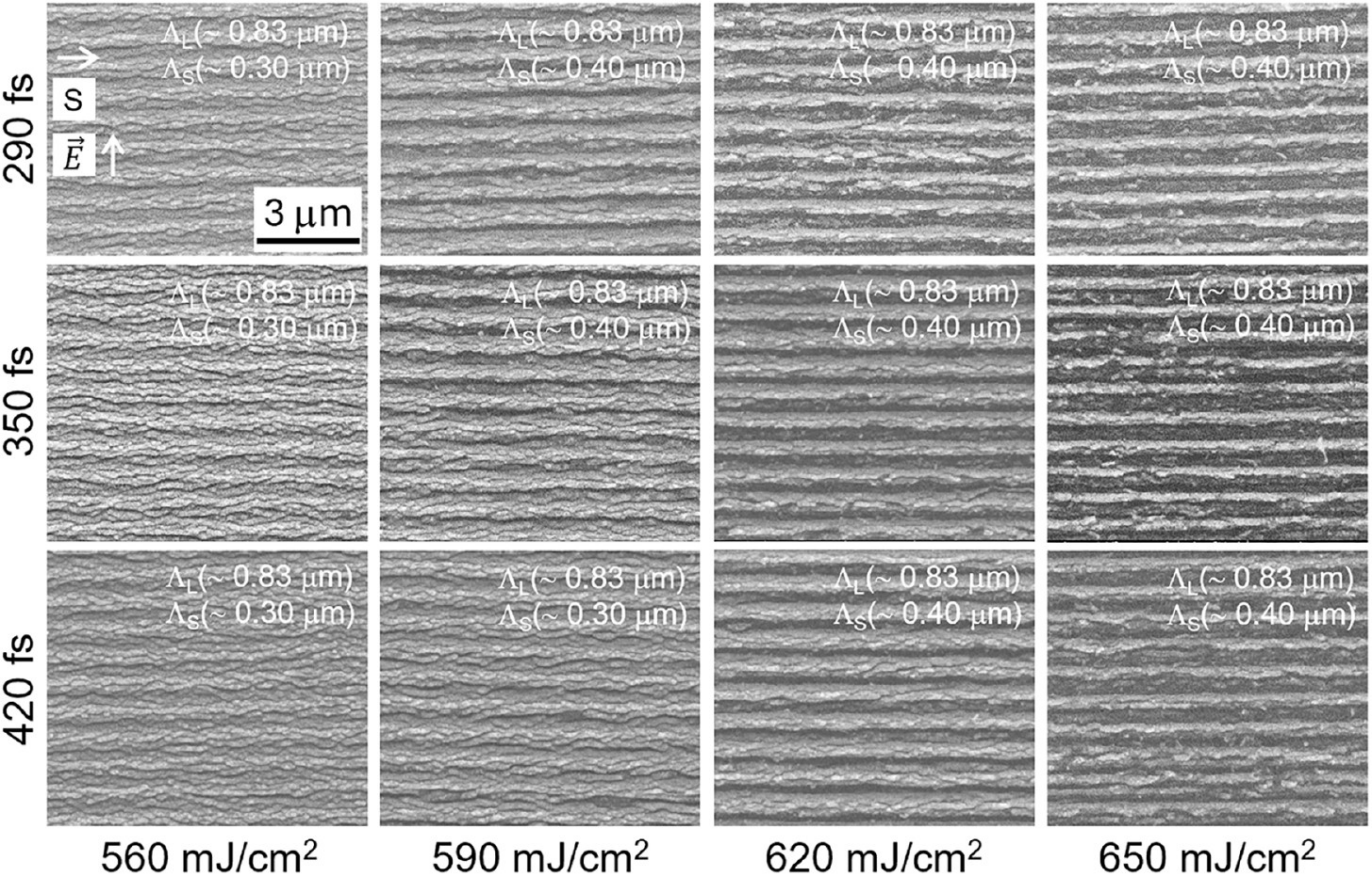
SEM images at the same magnification of FTO samples processed with fs-laser pulses at the indicated pulse duration and fluence. The arrows indicate the orientations of the polarization of the laser beam (E⃗) and the scan direction. Λ_L_ and Λ_S_ are the long (L) and short (S) periods of the structure calculated from the images.

For the intermediate pulse duration (350 fs), there is a broader fluence window where LSF-LIPSS structures with very clearly marked valleys can be achieved (590–620 mJ/cm^2^) without severe damage of the ridges. This suggests that electrical conductivity along the LIPSS axis should be preserved, while transverse conductivity must be reduced.

As in the case of fs-laser-processed ITO films,^29^ the presence of coherently propagated LIPSS is expected to generate a surface conductivity anisotropy. A schematic view of the LIPSS morphology and the way the electrical properties of the structured films were measured is given in Section 5.2. A more detailed discussion of this aspect can be found in ref. 29. Figure 4a–d illustrates the observed electrical anisotropy evaluated by four-point probing^50^ in a microscopic configuration for a film processed at 618 mJ/cm^2^ and a pulse duration of 350 fs. The figure includes the *I*–*V* curves (Figure 4d) measured for the microprobes aligned parallel or transversal to the LIPSS. The plot corresponds to an average of four measurements at different locations in the laser-processed film. From the values of the *I*–*V* curves and the distance between probes it is possible to estimate the effective microscopic sheet resistance parallel to (*R*_□||micro_ = 2.2 × 10^4^ Ω/□) or transverse (*R*_□⊥micro_= 4.30 × 10^5^ Ω/□) to the LIPSS, which corresponds to an anisotropy resistivity factor ξ_micro_ = (*R*_□⊥micro_/*R*_□||micro_) ≈ 20. The macroscopic sheet resistance of the processed material using a macroscopic four-point setup was similarly measured to confirm the quality of the coherently propagated structures. This was done over the same 6 × 6 mm^2^ processed region that was previously isolated from the pristine material by drawing an ablated square frame with the laser around the processed region. The measured values (*R*_□||macro_ = 5.7 × 10^4^ Ω/□, *R*_□⊥macro_ = 2.7 × 10^6^ Ω/□) provide a macroscopic anisotropy resistivity factor of ξ_macro_ ≈ 50.

**Figure 4:**
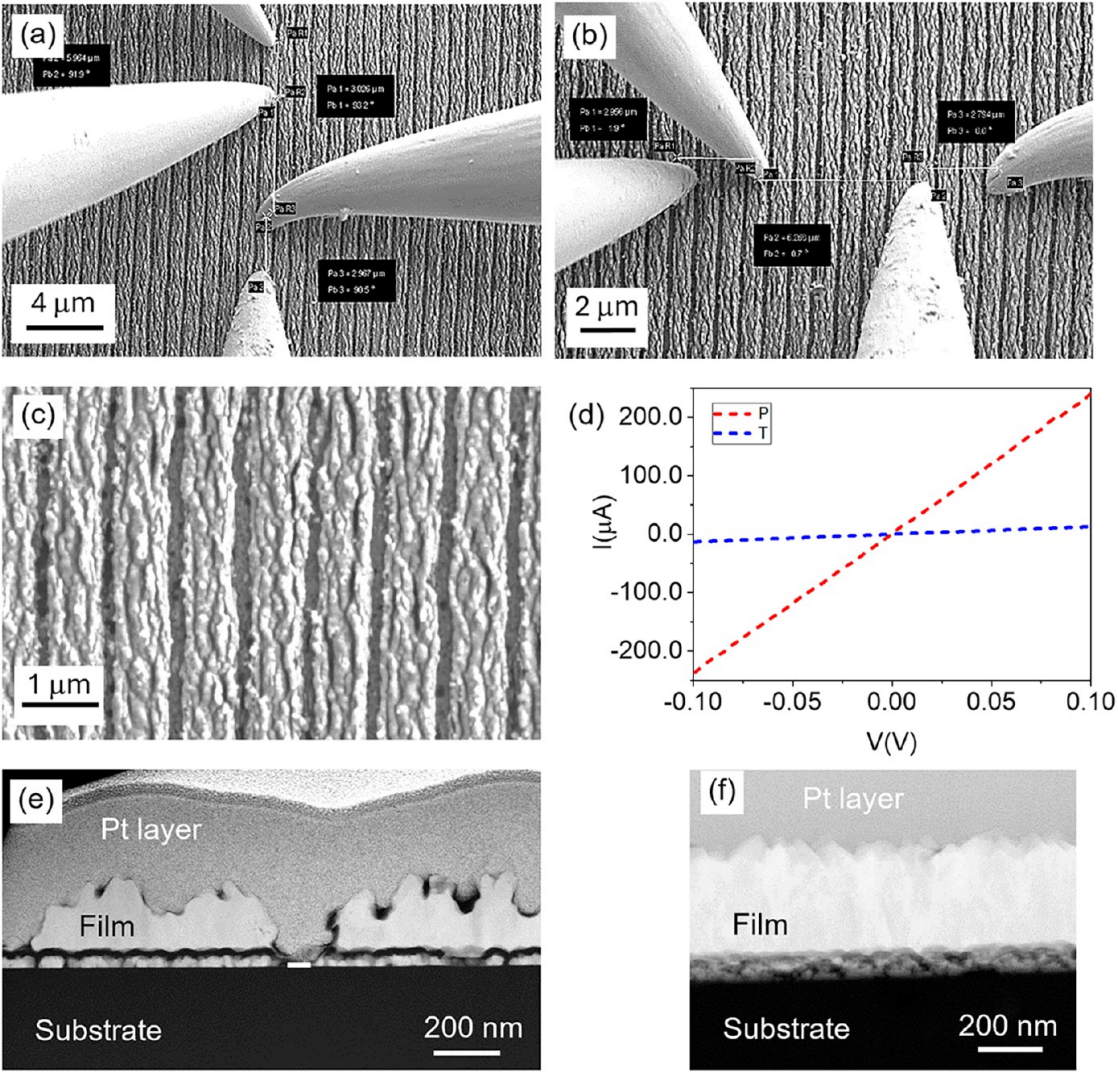
(a, b) SEM images during four-point probing measurements (microprobe configuration) in TEC-15 samples processed with 350 fs-laser pulses at 500 kHz at a fluence of 618 mJ/cm^2^. The images correspond to a measurement configuration parallel (a) or transversal (b) to the LIPSS axis (notice the different magnification of both images). (c) Shows a higher magnification image of the LIPSS-covered surface. (d) *I*–*V* curves for both probe orientations, parallel (*P*) (red dashed line) or transversal (*T*) (blue dashed line) to the LIPSS axis. (e, f) Scanning Transmission Electron Microscopy (STEM) image of a lamellae generated by standard Focused Ion Beam (FIB) sectioning (e) of a film processed at 620 mJ/cm^2^ and cryogenic FIB (f) of a pristine TEC-15 samples.

The close similarity of *R*_□||macro_ and *R*_□||micro_ values is a good indication of the excellent quality of the propagated structures. Nevertheless, *R*_□⊥macro_ is much higher than *R*_□⊥micro_. This is not surprising since, as can be seen in Figure 4c, the material remaining at the valleys, responsible for the low material conductivity transverse to the LIPSS, is very scarce. This may lead to some local variability in the microscale but would render a much smaller electron conduction contribution when averaged over a large surface. A precise determination of the resistivity (ρ) in both directions would require a precise value for the thickness of the ridges and a more elaborate model^51^ like the one used by Lopez-Santos et al. in previous work.^29^

A STEM cross-sectional image of a region processed with a very similar fluence in a different sample can be seen in Figure 4e showing that, as indicated, the period of the LSF-LIPSS is around 0.8 μm. The material remaining at the ridge has a thickness of around 200–250 nm and its contrast suggests that it is polycrystalline. Figure 4c and e clearly shows that the ridges are not flat and show complex worm-like features most likely related to the composition/phases present after material resolidification. These worm-like features are also evident in Figure 3 and lead to LIPSS splitting and strong ablation at the ridges upon irradiation at sufficient fluence. The figure also shows that the film has been nearly completely removed at the valleys of the structure, consistent with the much-reduced conductivity observed in the direction transverse to the LIPSS.

For comparison we have included in Figure 4f a cross-section STEM image of a pristine TEC-15 film. The observed thickness is ≈350 nm and the pristine material looks polycrystalline. Interestingly, a gray transition region with a thickness about ∼90 nm can be appreciated between the glass substrate (dark contrast) and the film (bright contrast). Energy Dispersive X-ray microanalysis (EDX) measurements show that the transition region is formed by Sn (21.2 at. %), Si (18.2 at. %), O (58 at. %), and Na (2.4 at. %). Such a Si-enriched layer composition is likely associated with the early stage of film formation during the APCVD process.

Globally, Figure 4c and e confirms that it is feasible to generate coherently propagated LIPSS, with very deep valleys paying a not so strong penalty in terms of thickness diminution at the ridges. For an adequate fluence interval it is thus possible to generate large electrical anisotropies in the processed surfaces as shown in Figure 4d. Assuming a ridge thickness of ∼250 nm, the resistivity of the material remaining in the processed surface can be estimated (from the *R*_□||micro_ measurement) to be ρ_P_ ≈ 5 × 10^–1^ Ω·cm. This value is a few hundred times higher than that of the pristine TEC-15. Again, this is not surprising since the resistivity of the laser-structured surface along and across the LIPSS axis is given by the depth and width of the ablated material and by the cross-section, composition, and structure of the material remaining at the ridges. We will show in Section 2.3 that the electrical performance of the laser-processed surfaces can be optimized through the laser processing parameters.

For what concerns the optical properties of the laser-processed regions, in the indicated fluence interval, the overall high optical transmission region is preserved and shifted to the IR. This can be appreciated in Figure 5 that shows the optical transmission spectra of a region irradiated with 420 fs laser pulses at 655 mJ/cm^2^ for the illumination beam polarization oriented parallel or perpendicular to the LIPSS. The overall behavior of the material response can be described in terms of the gap of SnO_2_ ^52^ and the free carrier density at room temperature generated by F-doping.^10^ The gap of SnO_2_ is ∼3.6 eV which confers FTO its transparency in the UV–vis spectral region beyond ∼340 nm, as it can be seen in the reference spectrum of nonirradiated FTO (black line in Figure 5). The presence of F-doping gives rise to the substitution of oxygen atoms generating free carriers at room temperature that generate a characteristic absorption band for near-IR wavelengths. In particular, the untreated FTO film shows a carrier density at room temperature (experimentally measured as described in Section 5) of 4.6 × 10^20^ cm^–3^, consistent with typical values observed in high temperature deposited APCVD and spray pyrolysis films.^53^ Such a carrier density generates a plasma cutoff wavelength around 1.6 μm, above which the material is strongly absorbent.

**Figure 5:**
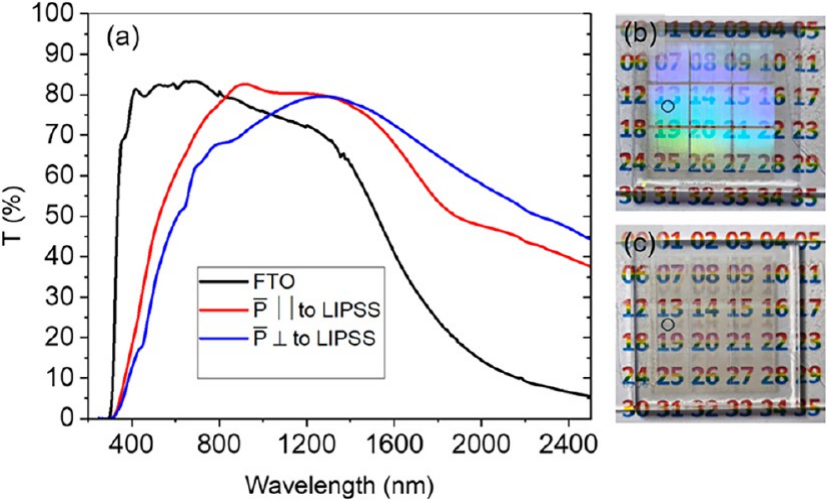
(a) Transmission spectra of a TEC-15 film region irradiated with 420 fs laser pulses at 655 mJ/cm^2^ for the illumination beam polarization oriented parallel (red) or perpendicular (blue) to the LIPSS. The spectrum of the nonirradiated surface (black) is included for comparison. (b, c) White unpolarized-light illumination images of a colored number sequence through several 6 × 6 mm^2^ regions processed with different fluences with oblique illumination (b) and nearly normal illumination (c). The position of the region processed at 655 mJ/cm^2^ is marked with a small black circle. Notice that the images do not have the same magnification.

The optical response of the irradiated material shows several characteristic features. First, a diminution of transparency for wavelengths below ∼650 nm due to scattering at the processed surfaces^54^ can be appreciated for both polarizations. Still, the transparency of the processed material at visible wavelengths for unpolarized light is very high, even in the presence of the scattering caused by the LIPSS structures. This can be clearly appreciated in the images included. They correspond to white-light illumination photographs of a colored number sequence through a processed film with nine areas of 6 × 6 mm^2^ irradiated at different fluences. The upper one shows some diffractive coloring in reflection, while the lower one, taken with nearly normal-incidence illumination to suppress diffraction effects, enables to appreciate the transparency of the irradiated and nonirradiated regions of the sample.

The second characteristic feature is the transmission increase compared to the pristine material for wavelengths beyond ∼1.2 μm. This would be consistent with a diminution of the free carrier density due to either a modification of the composition (F-content changes) or the structure (partial amorphization or grain size reduction) of the material remaining in the processed film, mainly at the ridges of the LIPSS. A decrease of the reflectance of the processed surface in this wavelength interval may also contribute to the observed transmission increase. Third, the IR transmission of the processed film is lower when the polarization of the illumination light is parallel to the LIPSS, indicating a clear, although not very strong form-birefringence effect.

### 2.2 Evolution of the F-Content in the Laser-Processed Regions

Given the direct relation between the F-content and the electrical conductivity of FTO films, no matter the synthesis method used,^10,47,55,56^ we have analyzed the evolution of the F-content in the laser-processed regions. It must be noticed though that a precise determination of F, especially in thin films, is a difficult task due to the light nature of F and its low cross-section for many assessment techniques based on electrons and ion probes. Besides, the atomic content of F is small in most applications where FTO is used as a TCO.^55^ In addition, there is a large dispersion in the F-content values reported in the literature on TCOs, likely related to the techniques employed for the analysis, with an F/Sn (atomic ratio) typically in the 0.02–0.15 range, the lower estimates usually being associated with X-ray photoelectron spectroscopy (XPS) measurements. F also shows a strong mobility and is easily removed upon electron or ion excitation.^57^ We therefore analyzed the evolution of the F-content using four different techniques: High-Angle Annular Dark-Field imaging (HAADF) in FIB-sliced samples in a STEM equipped with an EDX system operating at 200 kV, Nuclear Reaction Analysis (NRA) combined with Rutherford Backscattering Spectrometry (RBS), EDX in a conventional SEM and XPS measurements.

Figure 6a shows an STEM image of a FIB lamella in a sample processed with 350 fs laser pulses at 500 kHz and 618 mJ/cm^2^. The image corresponds to the ridge region at the left of the image shown in Figure 4e but at a higher magnification. The polycrystalline nature of the material present at the ridge can now be more clearly appreciated. Figure 6b–d corresponds to compositional maps showing the spatial distribution of other elements (O, Sn, and Si) obtained from the EDX analysis. While O appears nearly homogeneously distributed throughout the substrate and the film (Figure 6b), the Sn distribution shows a depleted region at the base of the ridge (Figure 6c) that is dominated by Si (Figure 6d). This Si-rich layer seems to be related to the Si-enriched transition region in the pristine film above-described (Figure 4f), although some Si segregation at the transition region because of laser irradiation cannot be discarded.

**Figure 6:**
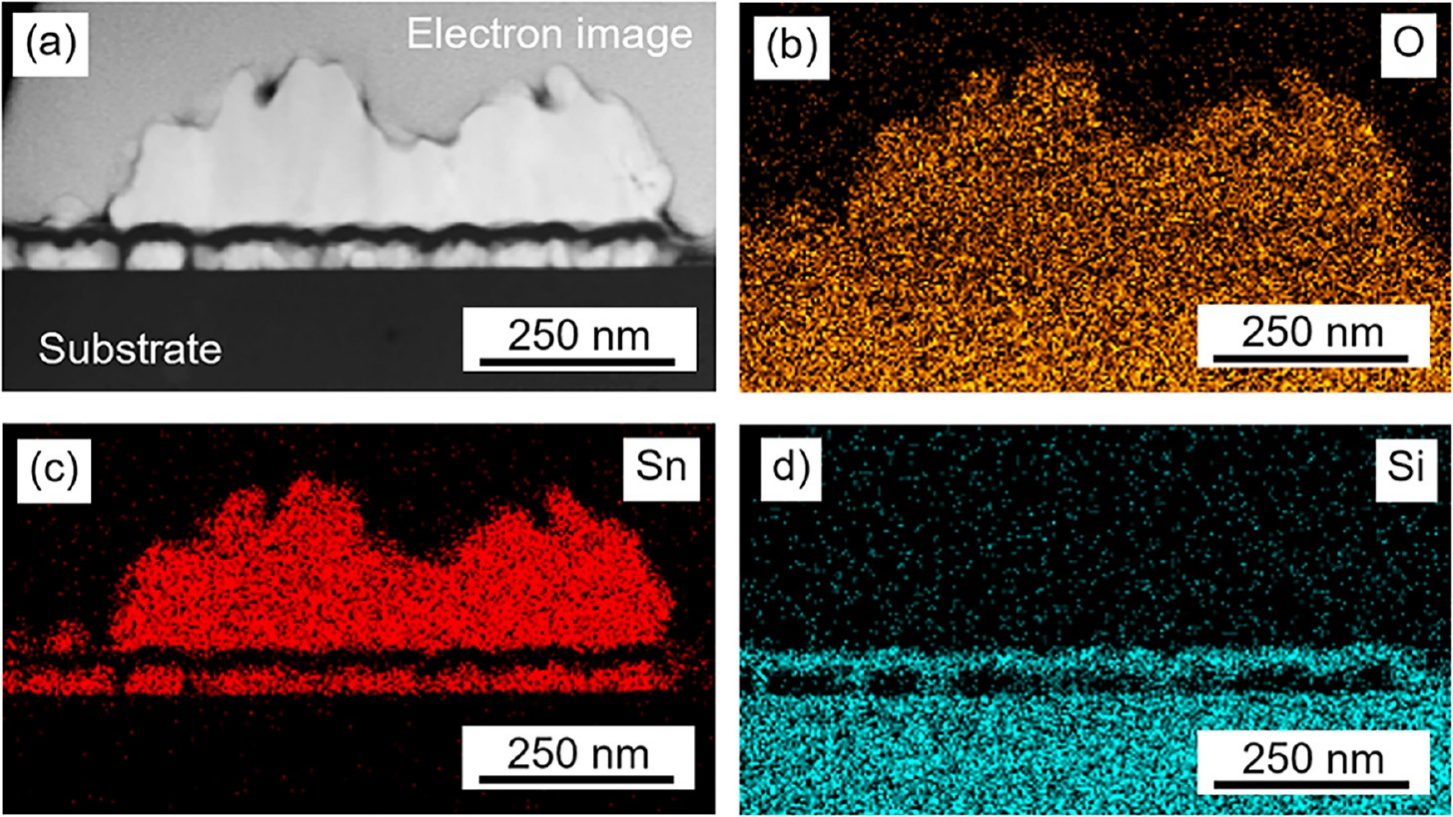
(a) STEM image of a ridge lamella generated by standard FIB sectioning of a TEC-15 film irradiated with 500 kHz laser pulses at 618 mJ/cm^2^. (b–d) EDX images of the ridge region corresponding to the spatial distributions of: O (b), Sn (c), and Si (d).

Interestingly, no trace of F was detected in any of the EDX spectra performed in FIB lamellae under standard or cryogenic conditions in pristine or irradiated films, which strongly supports that F was completely removed in the lamellas by the Ga^3+^ ion bombardment (50 keV). The removal of F due to ion bombardment was confirmed by planar EDX measurements (in a SEM) in the same samples but in areas far from the FIB-affected zone, where measurable amounts of F could be detected and quantified, in agreement with the observations reported in ref. 57. The composition of the near-surface region in pristine and laser-treated samples was also analyzed by X-ray photoelectron spectroscopy (XPS) but the F 1s peak (∼ 686 eV) was not observed in any case. Further information regarding the XPS measurements is given in the Supporting Information File (SIF).

The evolution of F and Sn concentrations as a function of laser fluence was similarly studied using RBS-NRA measurements in regions processed with 420 fs laser pulses, for which the surface morphology evolution with fluence looks smoother (see Figure 3, third row). Figure 7 shows several RBS spectra corresponding to the pristine sample and electrically isolated regions of 6 × 6 mm^2^ irradiated with the indicated fluences in the 620–680 mJ/cm^2^ interval. The RBS spectra of the laser-processed regions show a progressive diminution of the Sn content, related to the spatially averaged thickness diminution of the films and their increasing roughness, partly caused by the presence of LIPSS (see the Supporting Information File) for further information in this respect).

**Figure 7:**
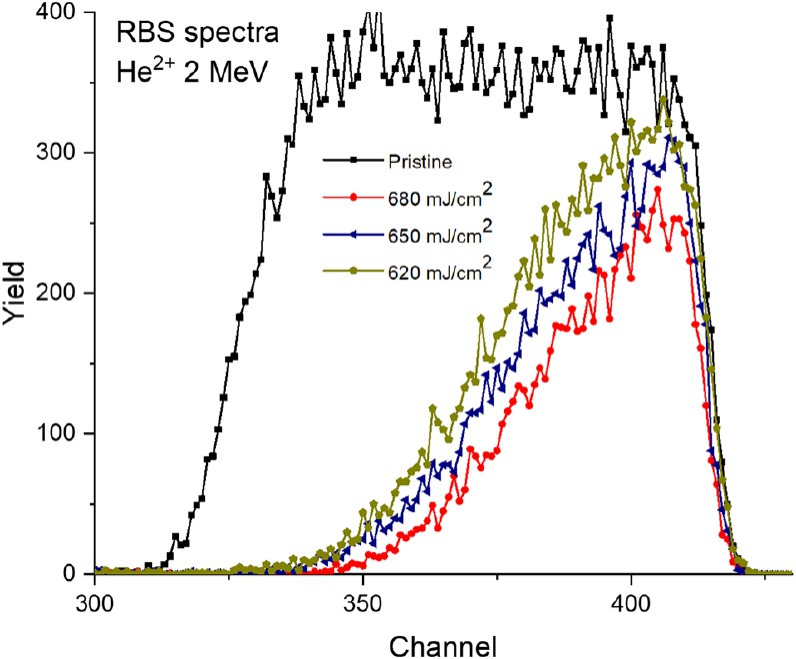
Sn signal from RBS spectra of TEC-15 FTO films. The spectra correspond to the pristine material (black line-and-symbols) and the material irradiated at the indicated laser fluences for a pulse duration of 420 fs.

The measured macroscopic resistivities along and across the LIPSS as a function of laser fluence for 420 fs laser pulses are included in Table 1 below. It can be clearly seen that both *R* **_□_**_∥macro_ and *R* **_□_**_⊥macro_ show an increasing behavior. In the case of *R* **_□_**_⊥macro_ the increase is caused by the larger depth of the valleys of the LIPSS, while the increase of *R* **_□_**_∥macro_ relates to the spatially averaged thickness diminution (consistently with the combined RBS and NRA analysis shown in the SIF) and the expected compositional changes in the ridges of the LIPSS. The observed trend of increase of *R* **_□_**_∥macro_ and *R* **_□_**_⊥macro_ indicates that the depth of the valleys strongly increases with fluence, while the thickness of the ridges decreases too, but at a lower rate, something which is clear from the evolution of ξ_macro_ with fluence.

**Table 1:** Macroscopic Resistivities for Areas Processed with 420 fs Laser Pulses^a^

Fluence [mJ/cm^2^]	*R*_□||macro_ (kΩ/□)	*R*_□⊥macro_ (kΩ/□)	ξ_macro_ (*R*_□⊥macro_ /*R*_□||macro_)
620	0.15	0.45	3
630	0.29	1.45	5
636	0.43	3.44	8
642	1.50	19.5	13
650	2.60	52.0	20
655	1.0	154.0	154
660	52.0	9256	178
667	22.0	14806	673
680	47.0	32994	702

a Macroscopic resistivities along (*R* **_□_**_∥macro_) and across (*R* **_□_**_⊥macro_) the LIPSS measured in TEC-15 films in 6 × 6 mm^2^ areas processed with 420 fs laser pulses at the indicated fluence (*f*_rep_ = 500 kHz, *d* = 4 μm, *v* = 2.5 m/s). The macroscopic electrical anisotropy factor (ξ_macro_) is also included.

The values in Table 1 also evidence that very large electrical anisotropies can be achieved for this pulse duration (420 fs) but with a large penalty in terms of increasing *R*_□∥macro_ above the value of the pristine material. As indicated above, for 350 fs there is a broader fluence window (590–620 mJ/cm^2^) where LSF-LIPSS structures with very clearly marked valleys can be achieved without severe damage of the ridges. Instead, for 420 fs pulses this only occurs for a fluence around 620 mJ/cm^2^ while severe damage at the ridges is already observed at 650 mJ/cm^2^ (see Figure 3). This explains the apparently anomalous behavior observed in the table data for some fluences above 650 mJ/cm^2^ (i.e., 655 and 660 mJ/cm^2^). Such anomalies are further discussed in Section 2.3.

Since RBS measurements are sensitive only to the Sn and (O + F) content, the F content was determined from NRA measurements. However, the F content determined by the combined use of NRA and RBS is extremely low in the pristine film, with an F doping estimated to be just around 1%. This value is well below typical values reported in the literature^10,56^ and well below the doping level here estimated by EDX (see below). This suggests again that ion bombardment during the RBS-NRA measurements leads to the removal of F atoms. This conclusion is further supported by the fact that in the whole fluence interval studied, the estimated F/Sn content derived from RBS-NRA measurements remains unchanged and shows a value similar to that of the pristine film (see the corresponding values at the SIF). Thus, it seems clear that F removal upon ion bombardment is the cause of the low F contents estimated by ion-beam-based techniques.

Consequently, we estimated the F-content in the laser-processed regions using electron-based interactions (EDX) following the approach described in ref. 56. For the F-content estimate it is important to avoid the contribution of elements present in the glass substrate (such as Si, Na, Mg, Ca, and Al) or those spuriously present at the surface (C). Additionally, the contribution of O from the substrate can lead to a strong underestimation of the elements present only in the film (Sn, F). We must indicate at this point that the use of a very low acceleration voltage (2 kV for instance) might reduce the depth interrogated by the e-beam. However, Sn is a relatively heavy element. Thus, its detection is not possible for such a low voltage. To detect the L_α_ X-ray line of Sn (∼3.4 keV), the acceleration voltage at the SEM needs to be at least above 7–8 kV and the electron beam interaction depth would reach the substrate. We therefore preferred to achieve a good signal from Sn and F and used an acceleration voltage of 15 kV. In addition, the normalization procedure considers only the contributions of Sn and F. The F-doping level in a given region of the film was calculated as

1 $$[F\left(doping\;in\;\%)\right]=\frac{100\times F\;\left(at.\;\%\right)}{Sn_{film}\;\left(at.\;\%\right)+O_{film}\;\left(at.\%\right)}$$

assuming that O (at. %)_film_ = 2 × Sn (at. %)_film_, which avoids any overcontribution of O from the substrate by assuming stoichiometry of the oxide in the film (stannic oxide, SnO_2_). Even when this assumption can be considered questionable in the irradiated material, the approach is better than adding a false contribution of O coming from the substrate, especially when the films get thinner because of laser irradiation. The error in the determination of Sn can be considered about 1 at. %, whereas in the case of F, given its low cross-section, we have assumed an error of 2.5 at. % (absolute) to ensure that the recorded variations are significant. Unless otherwise specified, composition measurements were averaged over a region of 4 × 4 μm^2^.

Figure 8a shows an SE-SEM image of a pristine TEC-15 film, whereas Figure 8b shows the EDX spectrum obtained over the framed region in Figure 8a, evidencing the presence of O, F, Si, and Sn in the interaction volume. The Si signal is caused by the glass substrate, while the O signal derives from the content of this element both at the substrate and the film. A semiquantitative estimate of the content of elements inside the interaction volume of the SEM e-beam has been made as shown in Table 2. It shows the values obtained considering all of the elements identified in the EDX spectrum (first row) as well as considering only the contributions of F and Sn (second row).

**Figure 8:**
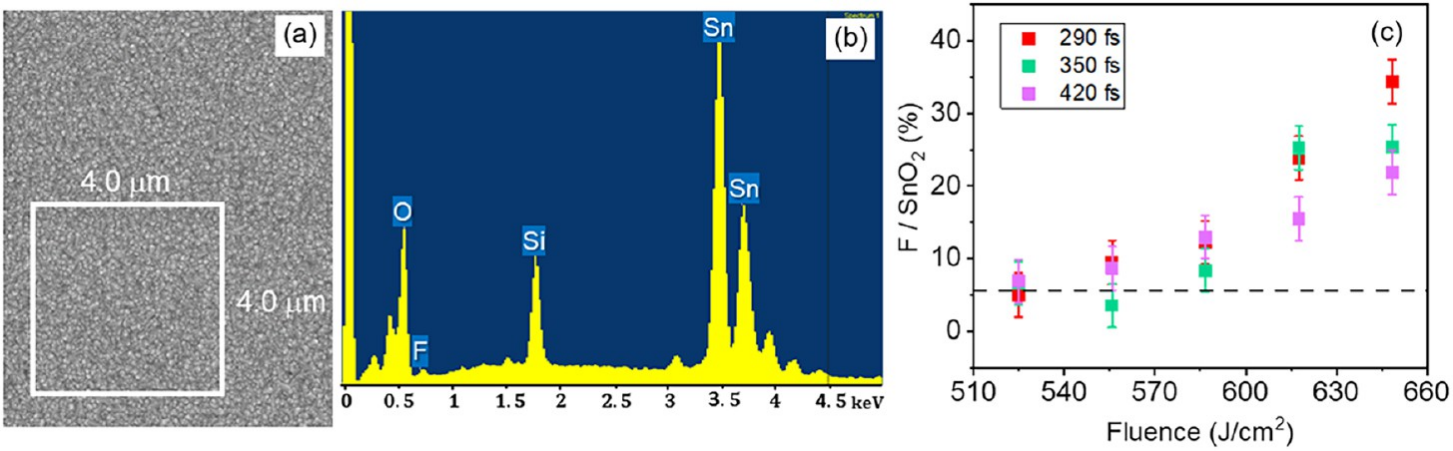
(a) SE-SEM image of a pristine TEC-15 film. The square indicates the region and scale where the EDX measurements were performed. (b) EDX spectrum at the region indicated in the SEM micrograph with the main peaks of elements identified: O, F, Si, and Sn. (c) F-content estimate (see text) in LIPSS regions in TEC-15 films for the laser pulse durations indicated. The measurements were averaged over several squared regions of 4 × 4 μm^2^ at the center of irradiated zones (1 × 1 mm^2^). The error bars correspond to an assumed F (at. %) error of 2.5 at. % (absolute). The level of the pristine material is indicated as a dashed black line, according to the data from Table 2.

**Table 2:** Elemental Quantification Estimates in the FTO Region Indicated in Figure 8 ^a^

	O	F	Si	Sn	F/Sn	F/SnO_2_ (%)
All elements (at. %)	75.1	–0.45	5.76	19.68	–0.023	–0.26
Only F and Sn (at. %)		14.7		85.3	0.17	5.7

a Values were derived from the EDX spectra considering all of the elements detected and the elements present only in the film.

From these values, it is clear that considering all the elements detected in the e-beam interaction volume leads to a strong underestimation of the F-content (which shows a negative value) and a strong overestimation of the amount of O, which should not exceed approximately 40 at. % in the film. This artifact is caused by the fact that at the e-beam acceleration potential used for imaging and EDX spectral data acquisition (15 kV), X-ray photons coming from the glass substrate are detected, as evidenced by the presence of Si and excessive oxygen in the analyzed spectrum. Considering only the F and Sn signals (the two elements present only in the film), the elementary concentration leads to an estimate of F doping of ∼ 5.7% (F/SnO_2_) using eq 1, which is in the order of typical values reported by many authors for high-performance FTO films produced by different techniques.^9,10,55,56^ We have thus considered the second approach to analyze the evolution of the F-content over regions of ∼4 × 4 μm^2^ at the center of the laser-processed areas (1 × 1 mm^2^). To ensure that the e-beam was not affecting the F-content in the irradiated regions^56^ several spectra were acquired in each processed region. Reproducibility has been assessed by iterative measurements. The final spectra recorded were acquired in less than 10 s with three iterations. Figure 8c shows the corresponding results for the [F/SnO_2_] estimates for laser-processed regions in the same conditions shown in Figure 3. However, we must emphasize that these data must not be considered as a precise determination of the F-content but are simply aimed at providing an overview of the relative changes in the F-content upon laser processing.

For the three pulse durations analyzed and laser fluences above ∼570 mJ/cm^2^ the F-content increases above the level of the pristine material beyond the error limit and shows a monotonically increasing behavior, which is faster for the shorter pulse duration (290 fs) and smoother for the longer one (420 fs). For a fluence of 590 mJ/cm^2^ the relative amount of F with respect to the pristine material is nearly doubled (close to 10%). The increase of F concentration for increasing fluences can be understood by the large binding energy difference between F and O with Sn: F 1s, 684.9 and 685.7 eV (for substitutional and interstitial positions) and O 1s, 530.9 eV.^9^ This difference points to a preferential release of oxygen with respect to fluorine when the material decomposes because of the large temperature rise caused by the laser, thus leading to the observed [F/SnO_2_] increase with fluence. Interestingly, for a given fluence above 600 mJ/cm^2^, the apparent oxygen preferential loss is higher for the shortest pulse duration and smaller for the longer one in agreement with observed morphological differences. The worm-like features observed in the ridges of the LIPSS (see for instance Figure 4c) also suggest that upon solidification some phase segregation occurs in which the F-excess might involve the formation/segregation of an F-enriched phase like SnF_4_. At the same time, as fluence is increased the melting and evaporation of the surface material lead to a progressive diminution of the film thickness both at the ridges and the valleys of the LIPSS structures and eventually to the formation of isolated lines leading to the already indicated strong electrical anisotropies (see Table 1). This can be clearly seen by comparing Figure 4f and e respectively corresponding to FIB lamellae cross-sections of a pristine sample and a region processed at 500 kHz with a laser fluence of 618 mJ/cm^2^ (*v* = 2.5 m/s, *d* = 4 μm)

### 2.3 Anisotropy Evolution as a Function of Fluence

Despite the difficulties in the assessment of the F content, our results prove that the generation and coherent propagation of LIPSS in FTO are feasible and controllable. To better understand the role played by film composition and morphology upon laser processing in the induced electrical anisotropies, we have further analyzed the evolution of the macroscopic electrical anisotropy with fluence for a pulse duration (350 fs), for which LSF-LIPSS leads to marked valleys over a broader fluence interval (590–620 mJ/cm^2^).

Figure 9 shows a plot with the evolution of the macroscopic anisotropy resistivity factor ξ_macro_ = (*R*_□⊥macro_ /*R*_□||macro_) as a function of fluence for 350 fs laser pulses. The results correspond to two different series of measurements performed on areas of 6 × 6 mm^2^ irradiated at the indicated fluence in two different TEC-15 samples. The values of ξ_macro_ show a similar trend for both irradiation series. For low fluences, below approximately 605 mJ/cm^2^, values of ξ_macro_ around ∼5–10 are typically observed. This is consistent with the observed morphologies (see Figure 3), where the valleys of the LIPSS structures are not sufficiently marked to generate a strong conductivity diminution in the transverse direction. At the same time the increase in the F-content in the material at the ridges remains moderate (values below 10 at. %, see Figure 8c). The dispersion of the ξ_macro_ measurements in this interval is relatively small with values of *R*_□||macro_ ≤ 520 Ω/□. Notice that the increase in the F-content in the ridges may accentuate up to some extent the observed resistivity increase with respect to the pristine material. As soon as the fluence is slightly increased above this value (605 mJ/cm^2^), there is a strong increase of ξ_macro_ to values exceeding 40 or even above 100, due to the shallower thickness of the material at the valleys, which strongly increases *R*_□⊥macro_. The region of maximal anisotropy factor lies in a narrow interval of approximately 10–15 mJ/cm^2^. After the maximum, ξ_macro_ shows a fast decrease to values around ∼10–20.

**Figure 9:**
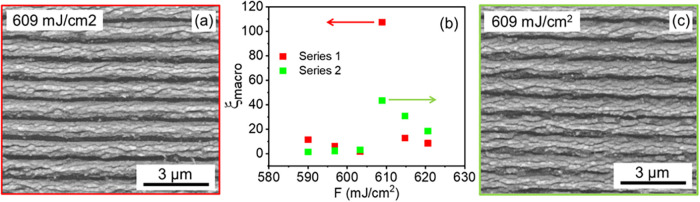
(a, c) SEM images of two areas processed at *F* = 609 mJ/cm^2^. (b) Macroscopic anisotropy resistivity factor (ξ_macro_) as a function of laser fluence in two series of measurements in different FTO samples (Series 1 and Series 2, red and green symbols, respectively) irradiated with 350 fs laser pulses (*f*_rep_ = 500 kHz, *d* = 4 μm, *v* = 2.5 m/s). The SEM images of the two areas correspond to Series 1 (red frame) and 2 (green frame), respectively. The arrows in Figure 9b point to the corresponding SEM micrograph in each series.

The large variability of ξ_macro_ in the region of the maximum for the two series of experiments performed is also appreciated in the subtle but appreciable differences in the SEM images of the two regions irradiated with the same parameters included in the figure. For the first irradiation series, at 609 mJ/cm^2^, the valleys of the structure (Figure 9a) are much more clearly defined than in the second series (Figure 9c) while the ridges look somewhat smoother. This is consistent with the *R*_□||macro_ and *R*_□⊥macro_ values measured in each case (*R*_□||macro_ = 2.3 kΩ/□ and *R*_□⊥macro_ = 247 kΩ/□ for the first series, whereas *R*_□||macro_ = 0.6 kΩ/□ and *R*_□⊥macro_ = 26 kΩ/□ for the second one). The higher value of *R*_□⊥macro_ indicates that the valleys nearly reach the substrate and the higher values of *R*_□||macro_ indicate the combined effect of a lower thickness at the ridges and the increased content of F-doping above the optimum.

However, the variability observable for very close processing conditions is intriguing: it is feasible to achieve values ξ_macro_ > 3500 with relatively low values of *R*_□||macro_. This is illustrated in Figure 10, which corresponds to a region irradiated at *F* = 618 mJ/cm^2^ in a different TEC-15 sample. The corresponding electrical measurement values are *R*_□||macro_ = 1 kΩ/□ and *R*_□⊥macro_ = 3.6 MΩ/□. The SEM image shows very neat and deep valleys and well-defined ridges. It is worth noting that *R*_□||macro_ is only a factor of 70 higher than that of the pristine TEC-15 film and thus remains within practical limits for device development. As discussed above, the LIPSS formation mechanism is given by the initially weak absorption of the laser wavelength by free carriers caused by the doping with F. Thus, the variability in the material response upon laser processing for very small changes of fluence (cf. Figure 10 vs Figure 9) strongly suggests the presence of local inhomogeneities in the coupling of laser energy to the films. These inhomogeneities could be related to the local distribution of F in the films on the microscale. Indeed, Chemical Vapor Deposition (CVD) or spray pyrolysis of FTO films delivers the reagents leading to the formation of droplets of different sizes onto a high-temperature substrate. These droplets and their subsequent reaction/decomposition are known to play a crucial role in the deposition process affecting the morphology and physical characteristics of the films.^55^

**Figure 10:**
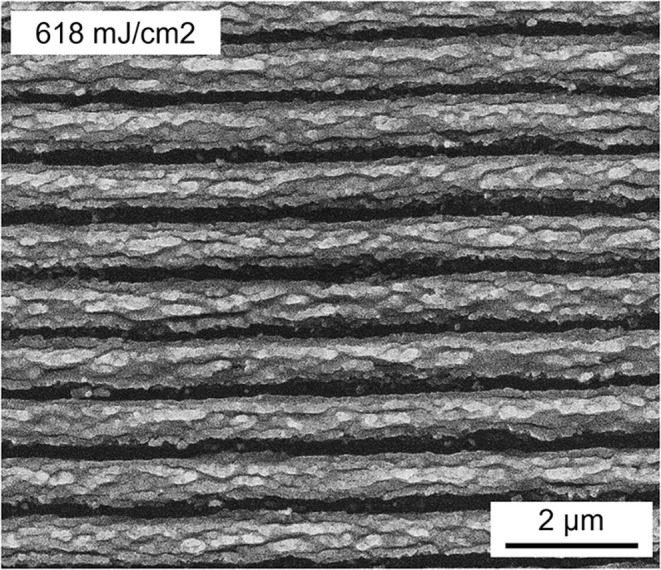
SEM micrograph of a TEC-15 film region processed with 350 fs laser pulses (*f*_rep_ = 500 kHz, *d* = 4 μm, *v* = 2.5 m/s, *F* = 618 mJ/cm^2^). The measured values of sheet resistance are *R*_□||macro_ = 1 kΩ/□ and *R*_□⊥macro_ = 3.6 MΩ/□, leading to ξ_macro_ = 3600.

To reinforce this hypothesis, we have investigated the local distribution of F in pristine TEC-15 films by using the EDX-based estimation approach described above. Two 25.4 × 25.4 mm^2^ samples were segmented in nine different regions of 6 × 6 mm^2^ and the F-doping level was determined by EDX measurements in regions of 4 × 4 μm^2^ at the center of each region using eq 1 (see Section 2.2 above). The result of these measurements is shown schematically in Figure 11, showing that there is a large local variability of the F-doping level in the microscale, most likely related to the deposition technique (APCVD). Measurements at random positions provided values similarly scattered: 6.0 at. %, 5.2 at. %, 3.5 at. %, 2.5 at. %, 4.7 at. %, and 0.24 at. %. The inhomogeneities in the F-content were also analyzed over regions of ≈80 × 100 μm^2^ showing variabilities of at least a factor of 2, typically in the range of 2% to 4%.

**Figure 11:**
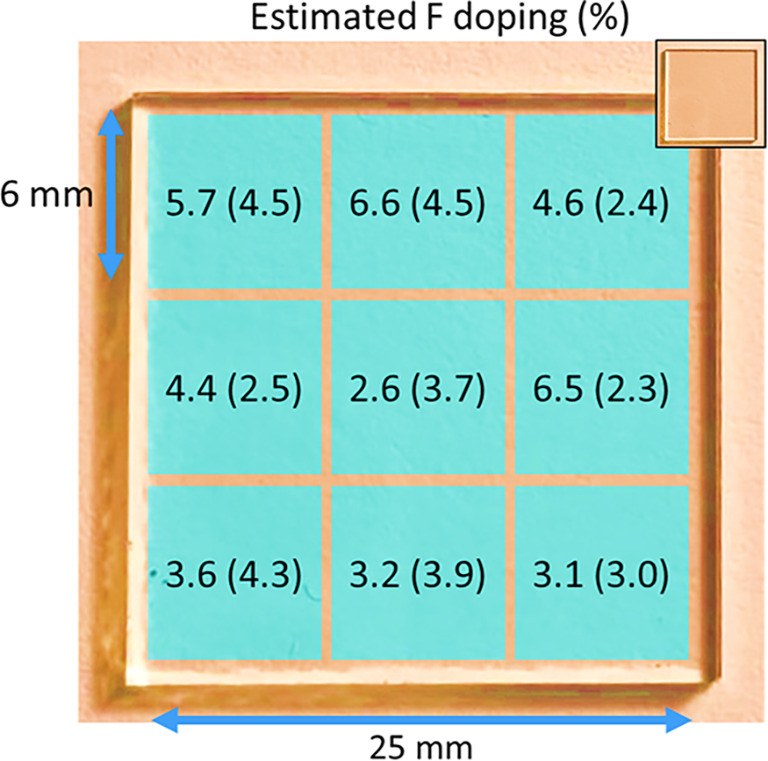
Estimated F-doping (%) at 9 different locations of a 25 × 25 mm^2^ TEC-15 sample. The sketch shows the location and size of the nine regions analyzed. The numbers in parentheses correspond to a similar measurement in a different sample using the same procedure. The measurements were performed using EDX measurements averaging over 4 × 4 μm^2^ regions at the center of each 6 × 6 mm^2^ region. The inset shows the piece of TEC-15 without the blue squares, to emphasize that the highlighted 9 areas are not laser-processed zones.

Therefore, it is expectable that we observe small differences in the amount of initial local absorption of the sample (notice that the focused laser beam dimensions are 2ω_0_ = 37 μm and the scan line separation *d* = 4 μm). This would affect the microscale morphology of the coherently propagated LIPSS, generating relatively small morphological differences for the same processing parameters that in turn can severely affect the final electrical anisotropy induced (cf. Figures 9 and 10). It must be emphasized that macroscopic measurements of the sheet resistance of the pristine FTO samples provide values consistent with the specification of the supplier (15 Ω/□). In any case, in spite the observed variability, we have been able to identify the optimal processing window in each sample and to achieve routinely macroscopic anisotropy factors above ∼10.

## 3 Challenges, Alternatives, and Potential Applications

The results presented confirm that the coherent propagation of LIPSS in FTO films over large areas is feasible and controllable through processing parameters. In particular, the processed structures can indeed show a very large electrical resistivity anisotropy. As described in Section 1, this can be useful for a range of practical applications such as transparent heaters, sensors, templates to produce aligned metal nanostructures, substrates for electrically controllable wettability, or the development of fluorescence anisotropies in perovskite films. The main issue for these laser-enabled large-area applications lies in the fluctuations of the material response caused by local inhomogeneities of the local F content of the film in the microscale. This problem, apparently related to the chemical deposition technique used in the commercial samples analyzed, can be circumvented using other preparation methods like reactive sputtering. Indeed, the use of sputtering for the growth of FTO films has been studied by many authors with excellent results,^56,58^ although the simplicity and lower cost of chemical deposition techniques at large scale have boosted methods such as APCVD or spray pyrolysis.

In the next subsection, we describe the feasibility of producing a laser-written transparent electrothermal deicing device based on the laser processing of TEC-15 films with moderate electrical anisotropy.

### 3.1 A Laser-Written Transparent Electrothermal Deicing Device Using Coherent LIPSS in a TEC-15 FTO Film

The electrothermal efficiency of the LIPSS-structured surfaces was evaluated by fabricating a laser-written, deicing device prototype in a TEC-15 FTO film. The goal is to take advantage of the electrical anisotropy induced by the coherent propagation of LIPSS to optimize the surface electrical charge transport. The device architecture is shown in Figure 12a. Specifically, an 8 × 8 mm^2^ active area was structured with LIPSS (yellow lines), oriented parallel to the electrode axis. The processing conditions were *f*_rep_ = 500 kHz, *d* = 4 μm, *v* = 2.5 m/s, and *F* = 621 mJ/cm^2^. The green regions denote the position of the electrodes attached to the film on nonprocessed areas. The whole structure is isolated from the rest of the film by laser-written ablated lines (in black color). To ensure efficient charge injection, 1 mm width nonprocessed FTO buffer regions were maintained between the LIPSS-structured region and the horizontal ablated lines allowing current to flow from one electrode to the other through the LIPSS ridges. For comparison, a similar device was built on a nonprocessed FTO film as sketched in Figure 12b.

**Figure 12:**
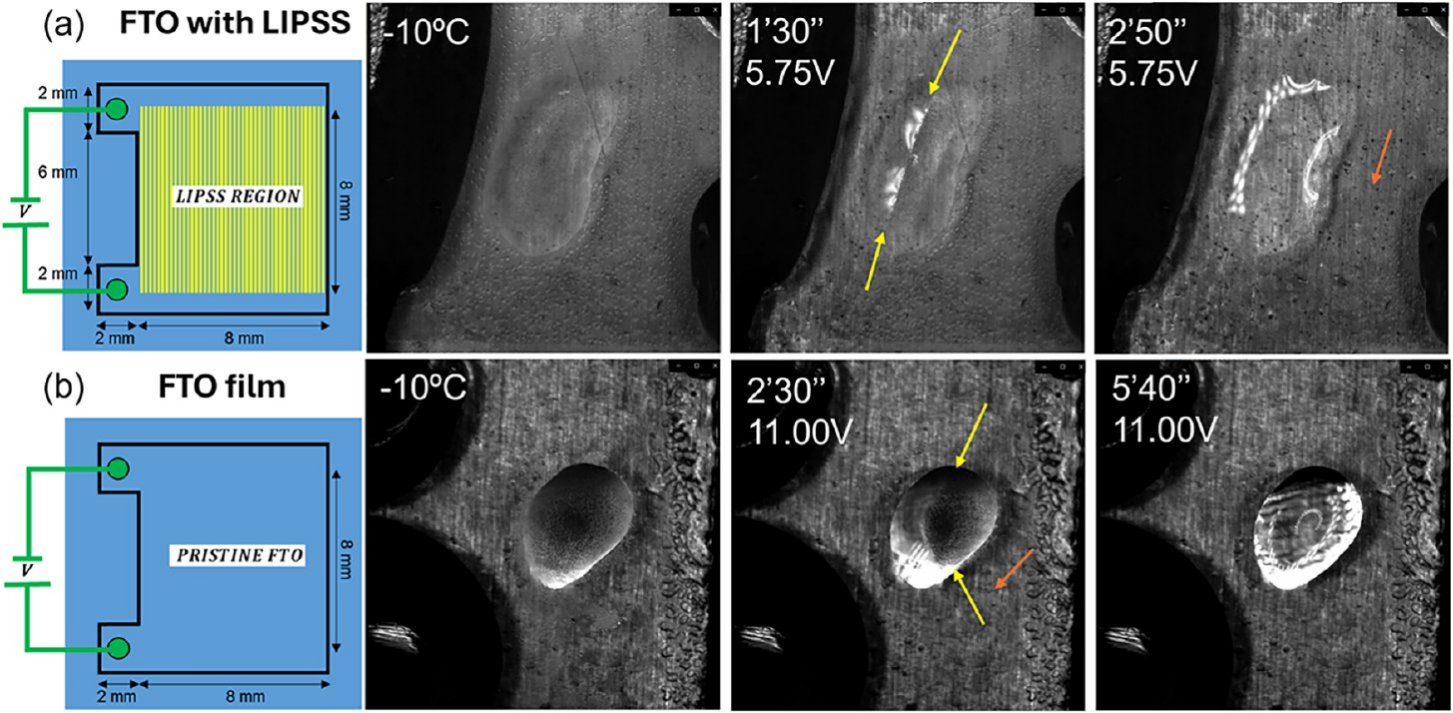
Schematic layout of the electrothermal devices including the electrical connections to an external voltage source for the LIPSS-processed (a) and pristine TEC-15 (b) samples; sequence of images corresponding to different stages of the induced deicing phenomena (upper row, LIPSS-processed sample; lower row, pristine TEC-15): (left to right) before, during, and after the application of the corresponding electrical voltage needed to promote the ice particle melting until the complete deicing process. Yellow arrows point to the advance of the thawing front, whereas orange arrows indicate the disappearance of frozen microdroplets caused by moisture condensation.

The device was placed in a temperature-controlled chamber with a 1 μL droplet of Milli-Q water deposited on its surface at room temperature. The entire system was cooled down to −10 °C, when the surface stabilized to induce the water droplet’s freezing. This can be seen in the image in Figure 12 (−10 °C, upper row), where the frozen water droplet on the LIPSS-structured surface is opaque and exhibits an anisotropic wetting morphology unlike the isotropic, quasi-spherical morphology observed on pristine FTO (lower row). The temperature distribution can be seen in the thermographic map included in Figure S4 a1 of the SIF. The laser-induced multiscale roughness in the LIPSS-covered region promoted uniform microcondensation and subsequent freezing across the functionalized area, whereas frost formation on untreated FTO remained stochastic and localized.

A voltage of 5.75 V was then applied. This value was previously determined as the minimum value from which the defrosting process started. The advance of the deicing front is marked with yellow arrows in the image after 1′30″ in Figure 12, upper row. The front is parallel to the direction of the LIPSS following from the place where the electrical contacts were located, that is, vertically in the image.

As shown in the upper row of Figure 12, the deicing of the deposited water droplet was completed after 2′50″ when the droplet gets transparent. Moreover, the disappearance of frozen microdroplets (orange arrow pointing to a region that previously had them) cleaning the surface of previous moisture condensation is noticeable. At the point of full ice macroparticle melting on the LIPSS surface, the thermographic map identified a temperature increase of almost 40 °C (Figure S3 a2 in the SIF). In contrast, on the pristine TEC-15 FTO surface, random ice microparticles disappeared in the first moments of applied voltage as a sign of the uniformity of their distribution (Figure 12, lower row, 2′30″), with defrosting being more effective depending on the smaller size of the frozen droplets. Furthermore, for melting the ice macroparticle via the thermoelectric effect, the untreated FTO surface required an applied voltage of 11 V (Figure 12 lower row) and more than twice the processing time compared to the LIPSS-structured surface. Quantitatively, to maintain a comparable thermal gradient across an apparent area of 72 mm^2^, the pristine FTO sample required a power consumption of 2.09 W. In contrast, the LIPSS-structured surface achieved superior deicing performance with only 1.25 W. This 40% reduction in power consumption demonstrates that the laser-induced confinement of current density within the LIPSS ridges significantly enhances the electro-thermal conversion efficiency (*P* = *V* ^2^/*R*), leveraging the increased surface-area-to-volume ratio and the anisotropic resistivity of the nanostructured film.

Previous works on electrothermal systems based on transparent conductive oxides such as ITO have reported deicing voltages for continuous ITO films typically ranging between 10 and 20 V, with power densities on the order of 2–5 W cm^–2^, depending on thickness and sheet resistance of the film.^59,60^ Thus, the demonstrated performance of the LIPSS-structured FTO films as electrothermal devices is closer to other current alternative approaches based on percolating networks of silver nanowires, graphene films, 2D materials or dielectric/metal multilayers with rapid deicing at voltages below 5 V.^61–64^ However, those systems often face challenges related to long-term environmental stability, oxidation, adhesion, or mechanical durability. In contrast, FTO offers superior chemical robustness, thermal stability, and compatibility with large-scale industrial deposition processes. Importantly, the present strategy accommodates the surface morphology to tailor the in-plane resistivity response, thanks to a controlled redistribution of current density arising from the periodic modulation of surface resistivity induced by LIPSS. Here, such anisotropy is functionally exploited to direct the Joule heating front along a predefined axis, concentrating thermal power where needed and reducing overall energy consumption.

## 4 Conclusion

We have analyzed the formation and coherent propagation of LIPSS in FTO films under high-repetition-rate IR fs-laser irradiation aiming at the generation of electrical anisotropies for macroscopic applications. The results demonstrate that it is feasible to induce very large anisotropies with resistivities transverse to the LIPSS orientation that can be several orders of magnitude higher than the ones in the direction parallel to the LIPSS over large macroscopic areas. At the same time, the penalty in terms of the resistivity increase along the direction parallel to the LIPSS is relatively small. This implies that several large-area applications of coherently propagated LIPSS in FTO films are feasible, as shown in the case of a laser-written deicing device that we have produced using relatively small anisotropy values. We have also correlated the overall electrical properties of the generated structures with the morphology and compositional changes induced by the laser and unveiled the crucial role of the dynamic evolution of the F content during the process.

Surprisingly, the film response is conditioned by the film preparation procedure (APCVD) since it conditions the microscopic distribution of F and thus the initial local coupling of the focused laser beam with the film. Consequently, the microscale morphology of the coherently propagated LIPSS can be affected, generating relatively small differences for the same processing parameters that, in turn, can severely affect the final electrical anisotropy induced. Still, this issue could be circumvented using reactive sputtering deposition, enabling the development of the indicated large-area applications based on the coherent propagation of LIPSS in FTO films.

Finally, this work demonstrates that the accumulated fluence deposition at the valleys of the LIPSS enables a controlled in-plane electrical anisotropy that can be directly exploited for efficient electrothermal devices. Particularly, the engineered in-plane anisotropic resistivity promotes directional current flow and guided heat propagation of direct application for deicing purposes. The complete ice and water condensation removal has been resulted at significantly reduced voltage and power (≈1.25 W) compared to pristine FTO, with an energy saving of approximately 40%. Beyond enhancing electrothermal efficiency, this rapid prototyping approach preserves optical transparency, chemical robustness, and material simplicity, as it does not require multilayer stacks, hybrid composites, or additional functional coatings. These findings establish laser-induced anisotropic patterning as a powerful and scalable strategy for next-generation transparent heaters, offering improved energy management, spatial control of thermal fronts, and strong potential for applications in smart glazing, optical sensors, automotive systems, and aerospace anti-icing technologies.

## 5 Experimental Methods

### 5.1 FTO Samples and Laser Processing for LIPSS Generation

The FTO films used in this study consisted of commercial Atmospheric Pressure Chemical Vapor Deposition (APCVD) films (NSG TEC15) on soda-lime glass substrates. The films had ∼350 nm thickness and a maximum sheet resistance of 15 Ω/□. A detailed description of the morphology and properties of these films can be found in ref. 55. These commercial films are denominated as TEC-15 throughout the text.

Femtosecond laser processing of the films was performed in air using a Yb-doped amplified fiber laser (Satsuma HP^2^, Amplitude Systemes) operating at a central wavelength of 1030 nm with a maximum pulse repetition rate of 2 MHz. The minimum pulse duration of the system was 290 fs and could be tuned using the system compressor alignment. The laser beam was steered and conditioned using several elements to define its polarization and energy before reaching a galvanometric mirror scanner (ScanCube 14, Scanlab) equipped with an F-theta lens (*f* = 100 mm). A 5.5 mm-diameter pinhole was located at the input of the scanner in order to slightly increase the beam diameter at focus. The scanner provides a flat field, focusing area of 7 × 7 cm^2^ and was operated with commercial software (LaserDESK, Scanlab) to control the scanning parameters. Typically beam scanning was performed along the horizontal direction from the top to the bottom of the laser processed structure, with the beam polarization oriented vertically at the sample, and thus perpendicular to the scanning direction. The spot diameter of the Gaussian-shaped laser beam at the sample surface was determined, following the method proposed by Liu,^65^ to be 2ω_0_ = 37 μm (1/e^2^ intensity), where ω_0_ is the spot radius. The average power of the laser beam was determined with an Ophir 3A-PF-12 thermopile sensor with a sensitivity of 15 μJ and an accuracy of 0.2%. For a given average power of the laser, the energy of the individual pulses (*E*) and the local fluence pulse at the sample site was $F=2\cdot E/\left(\pi \cdot \omega_{0}^{2}\right)=2\cdot \mathrm{P̅}/\left(\pi \cdot \omega_{0}^{2}\cdot f_{rep}\right)$ , being *P̅* is the average power and *f*_rep_ is the repetition rate. With regards to the fluence values quoted in the manuscript it is worth noting that although the absolute error in the determination of local fluence is around 10 mJ/cm^2^, mostly related to the determination of the focused beam diameter and the transmission of the focusing optics (both fixed parameters), the comparison of fluences for different average power values of the laser enables to discriminate much smaller differences, around ±1 mJ/cm^2^.

Laser processing was used to texture the FTO film surfaces in square regions of various sizes, varying the laser repetition rate (*f*_rep_), pulse duration (τ), laser fluence (*F*), scan line separation (*d*), and scanning speed (*v*) to optimize LIPSS propagation and maximize their electrical anisotropy (difference in the sheet resistance measured parallel vs perpendicular to LIPSS orientation). The goal was to maintain a low resistivity parallel to the LIPSS, while significantly increasing transverse resistivity, ideally creating insulation. After an initial search of processing parameters, *f*_rep_, *d*, and *v* were fixed at 500 kHz, 4 μm, and 2.5 m/s respectively, which are the values used in the text unless otherwise specified.

### 5.2 Sample Characterization

Before and after processing, the fabricated microstructures were characterized in terms of morphology, composition, and optical and electrical properties. The macroscopic optical and electrical properties were assessed in large, squared areas (6 × 6 mm^2^) that were electrically isolated from the rest of the film by inscribing a deep laser-ablated line at high power after processing. Optical transmission (*T*) spectra were measured using a Varian Cary 5000 UV–vis–NIR spectrophotometer with a resolution of 1 nm over the 350–2500 nm range. To analyze the potential presence of optical birefringence upon laser processing, the same spectrophotometer was used with a polarization control element to illuminate the samples with light linearly polarized parallel to or perpendicular to the LIPSS orientation. In this approach, the collection of light was not modified, and therefore, the measurement was not total, and losses by scattering/diffraction cannot be discarded.

Morphology was assessed by Optical Microscopy (OM) and Scanning Electron Microscopy (SEM) in Secondary Electron detection mode (SE). OM characterization was performed with a Nikon Eclipse-Ti Microscope using illumination at 460 nm. The top-view images of the LIPSS structures were Fast Fourier Transform (FFT) analyzed to quantify the LIPSS period (Λ). For the SE-SEM characterization, an FEI Inspect-S SEM and a Jeol JEM 6500F FEG-SEM were used to get top-view, high-magnification images of the induced structures. Also, some of the pristine and laser-irradiated regions were sliced using a focused ion beam (FIB) of Ga^3+^ at 50 keV to produce thin lamellae both in standard and cryogenic conditions. These lamellae of the processed films were oriented transversally to the LIPSS and were subsequently inspected in a Titan Analytical STEM operated at 200 kV.

F- and Sn-contents of the pristine and irradiated samples were estimated by Energy Dispersive X-ray microanalysis (EDX) with the JEOL JEM 6500F FEG-SEM (equipped with an Oxford INCA Energy 200 microanalysis system) operated at an acceleration voltage of 15 kV. Since the irradiated surfaces are textured and this might lead to shadowing effects, we were extremely careful in preserving the orientation of the LIPSS at a given magnification during the EDX measurements, to make the measurements from different irradiated areas to be comparable. The methodology for quantification using the EDX measurements is discussed in the results section. In addition, the evolution of the fluorine content in the irradiated regions was analyzed by Nuclear Reaction Analysis (NRA) combined with Rutherford Backscattering Spectrometry (RBS). Sn- and O-contents were obtained by RBS using a He^2+^ beam at 1990 keV (1 mm diameter beam spot) at a scattering angle of θ = 165°. The F-content was measured through the ^19^F­(p, α_0_)^16^O nuclear reaction using an H^+^ beam at 1864 keV (1 mm diameter beam spot) with a scattering angle of θ = 150°. For both measurements the sample holder was tilted 7° to avoid possible channeling effects in the film or substrate. Compositional analysis was also performed in the FIB-sectioned lamellae using the above-indicated STEM microscope with a high-angle annular dark field (HAADF) detector for the acquisition of Z-contrast images, and an EDX microanalysis system. The composition of the near-surface region in pristine and laser-treated samples was also analyzed by X-ray photoelectron spectroscopy (XPS) in a SPECS spectrometer provided with a hemispherical analyzer (DLSEGD-PhoibosHsa3500), using nonmonochromatic Mg Kα radiation line to excite the spectra in a normal configuration. Data were recorded with a 50 eV constant pass energy mode for the general survey spectra and 30 eV for high-resolution spectra. These spectra were calibrated in binding energy using the C 1s photopeak associated with adventitious carbon surface contamination at 284.5 eV.

To facilitate an intuitive comparison of the morphologies induced and the electrical properties measured, we have sketched in Figure 13 the LIPSS morphology and how the four-point probing measurements were carried out. The coherently propagated LIPSS can be seen as a set of alternating bars of two different materials with different heights (H_1_, H_2_), widths (W_1_, W_2_), and resistivities (ρ_1_, ρ_2_) corresponding to the material at the ridges (H_1_, W_1_, ρ_1_) and at the valleys (H_2_, W_2_, ρ_2_) of the structure. Depending on the positions and orientation of the electrodes injecting the current for the electrical measurements, the effective electrical properties of the material parallel or transversal to the LIPSS were assessed.

**Figure 13:**
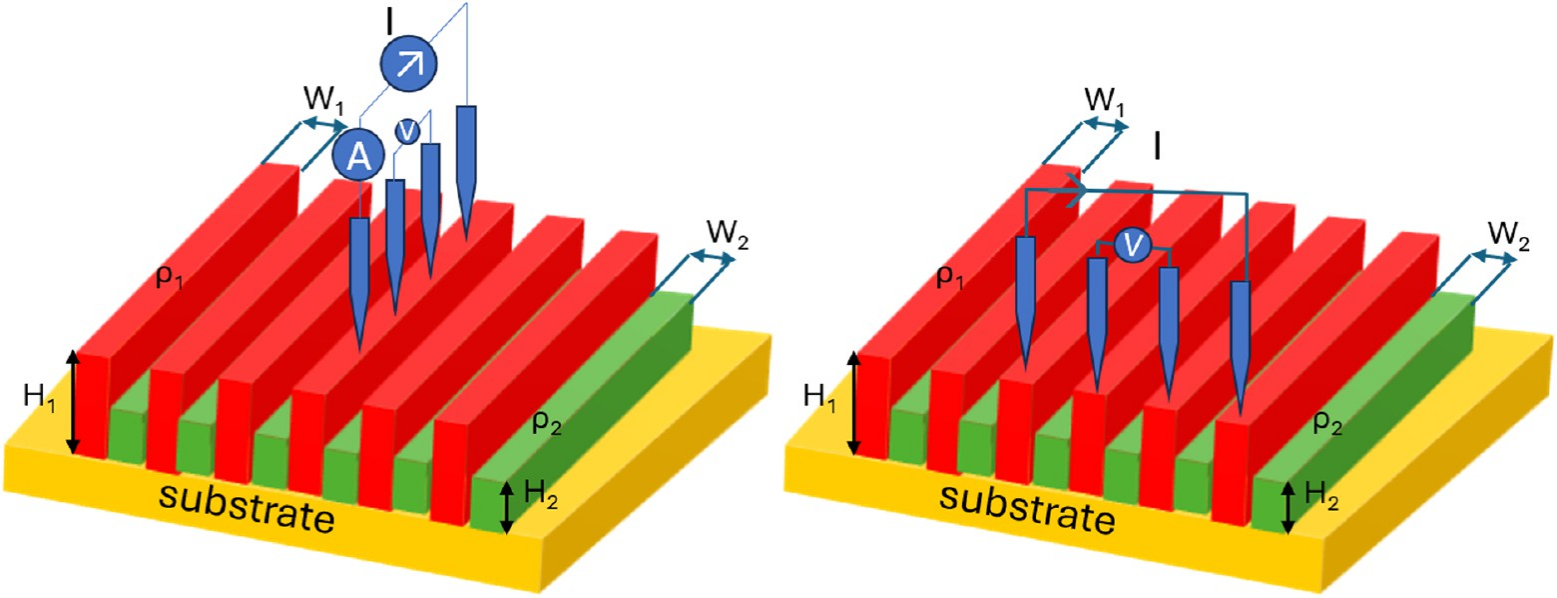
Schematic representation of the LIPSS structure including the material present at the ridges (red colored) and the valleys (green colored) with their characteristic heights (H_1_, H_2_), widths (W_1_, W_2_), and resistivities (ρ_1_, ρ_2_). The assessment of the electrical properties of the structures can be done with electrodes (blue colored) oriented either parallel (left image) or transversally to the LIPSS axis.

The electrical transport characterization of the pristine samples was performed at RT in the van der Pauw configuration by means of sheet resistance and Hall measurements in an ECOPIA HMS-7000. To quantify the electrical anisotropy of the processed areas, sheet resistance measurements were performed using a 4-Point-Probe Ossila device. Depending on how the tips are placed, parallel or transversal to the LIPSS orientation, we have determined two different values of sheet resistance, namely *R*_□||macro_ and *R*_□⊥macro_. The electrical anisotropy factor (ξ_macro_) was then calculated as ξ_macro_ = *R*_□⊥macro_ /*R*_□||macro_. Additional microscale electrical measurements were performed in a Zeiss Gemini 300 SEM microscope equipped with a Kleindiek micromanipulators system and an electron beam-induced current detector. For the four-point probe measurements, a set of Micro-Pico Probes T4–10 made of tungsten with a 3.3 mm length, 10 μm diameter, and <0.1 μm point radius was used. These measurements enable to determine the corresponding values of *R*_□||micro_ and *R*_□⊥micro_ along the directions parallel and perpendicular to the LIPSS and the corresponding electrical anisotropy factor, ξ_micro_ = *R*_□⊥micro_ /*R*_□||micro_.

The proof-of-concept laser-written transparent electrothermal deicing device has been fabricated in an 8 × 8 mm^2^ LIPSS-covered region adjacent to two untreated 2 × 2 mm^2^ regions located in the two adjacent corners of one side to make the electrical connections for the device (see Section 3.1). The whole assembly (LIPSS plus contacts) was electrically isolated from the rest of the film. The connections consisted of Cu wires attached to the FTO surface with Ag paint, covering the joints with a polymeric adhesive. A freezing test was performed with a 1 μL Milli-Q water droplet deposited on the surface of the LIPSS region at room temperature and cooled to −10 °C in a temperature-controlled chamber of a DataPhysics OCA 25 goniometer. Once the droplet was frozen, the thawing process induced by the electrothermal effect due to the application of a DC voltage to the contacts was monitored using the integrated top optical camera and a FLIR A65sc thermographic camera.

## Supplementary Material



## Notes

RBS, NRA, and XPS measurements performed in pristine and laser-processed samples as well as thermographic measurements performed (PDF)

## ○

Present Address: Centre of Membrane Separation, Adsorptions, Catalysis and Spectroscopy for Sustainable Solutions – KU Leuven, Celestijnenlaan 200F, BE-3001 Leuven, Belgium
