# Supplementary material for: Formation and Coherent Propagation of Femtosecond-Laser-Induced Periodic Surface Structures (LIPSS) in Fluorine-Doped Tin Oxide: Control, Potential Applications, and Challenges
Source: ACS Appl Mater Interfaces. 2026 Jun 22;18(25):36283–98. doi: 10.1021/acsami.6c06535 (PMC13339024; doi:10.1021/acsami.6c06535)
Supplement: Supplementary file 1 [file am6c06535_si_001.pdf]

Supporting Information

Formation and Coherent Propagation of Femtosecond-Laser-Induced Periodic Surface Structures (LIPSS) in Fluorine-doped Tin-Oxide: Control, Potential Applications, and Challenges

Gonzalo Gomez-Munoz[1], Rocio Ariza[1†], Fernando Nuñez-Galvez[2,3], Victor López-Flores[2,3], Fatima Cabello-Pardos[1], Belen Sotillo[4], Carlos Prieto[5], Jose Gonzalo[1], Francisco Javier Garcia-Lopez[6,7], Paloma Fernández Sánchez[4], Carmen Lopez-Santos[2,3], Javier Solis[1]*.*

[1]Instituto de Óptica-CSIC (IO-CSIC), c/Serrano 121, ES-28006 Madrid, Spain. [2]Instituto de Ciencia de Materiales de Sevilla-US-CSIC (ICMSE-US-CSIC), C/ Américo Vespucio 49, ES-41092 Seville, Spain. [3]Departamento de Física Aplicada I-Escuela Politécnica Superior, Universidad de Sevilla, c/ Virgen de África, ES-41013 Seville, Spain. [4]Departamento de Física de Materiales - UCM, Pl. de las Ciencias 1, ES-28040 Madrid, Spain. [5]Instituto de Ciencia de Materiales de Madrid-CSIC (ICMM-CSIC), Cantoblanco, ES-28049 Madrid, Spain. [6]Centro Nacional de Aceleradores, Av. Tomas. Edison 7. ES-41092 Seville, Spain, [7]Departamento de Física Atómica, Molecular y Nuclear. Universidad de Sevilla. ES-41012 Seville, Spain

Corresponding Authors: * gonzalo.gomez@io.cfmac.csic.es, j.solis@io.cfmac.csic.es

Present Addresses: † Centre of Membrane Separation, Adsorptions, Catalysis and Spectroscopy for Sustainable Solutions – KU Leuven, Celestijnenlaan 200F, BE-3001 Leuven, Belgium

Supporting Information Content

1) **Total thickness and roughness of the SnO_2:F layers measured by Rutherford Backscattering Spectrometry (RBS).**

RBS measurements were carried out in a pristine sample and in 9 different regions irradiated with increasing laser fluences from 620 to 680 mJ/cm^2, The RBS spectra were obtained using a He^{2+} beam at 1990 keV; with a PIPS silicon detector placed at an scattering angle, $\theta = 165^o$. The beam diameter was set to 1 mm. With these experimental conditions, the cross sections for all the elements in the sample are Rutherford type (i.e. $\sigma \sim Z^2$) and, therefore, the F signal is too small to be observed due to its low concentration and the overlap with the substrate signal. The spectra were analysed with the SIMNRA code. The goal of these experiments was to determine the thickness and depth distribution of the SnO_2 layers on the glass substrate, especially through the study of the Sn signal, which is completely separated from the rest of elements.

Figure S1 shows the experimental spectra corresponding to the pristine and irradiated regions with fluences of 620 and 680 mJ/cm^2, respectively. While in the pristine sample the Sn exhibits a constant concentration profile with depth, in the irradiated regions a significant amount of material has been removed, this deficit being greater as the laser fluence increases. Furthermore, in this case, the gradients shown in the Sn signals are indicative of layers with a high degree of roughness, in agreement with the TEM results. Table S1 summarizes the results of the RBS measurements, where for all regions the composition has been considered to correspond to SnO_2.

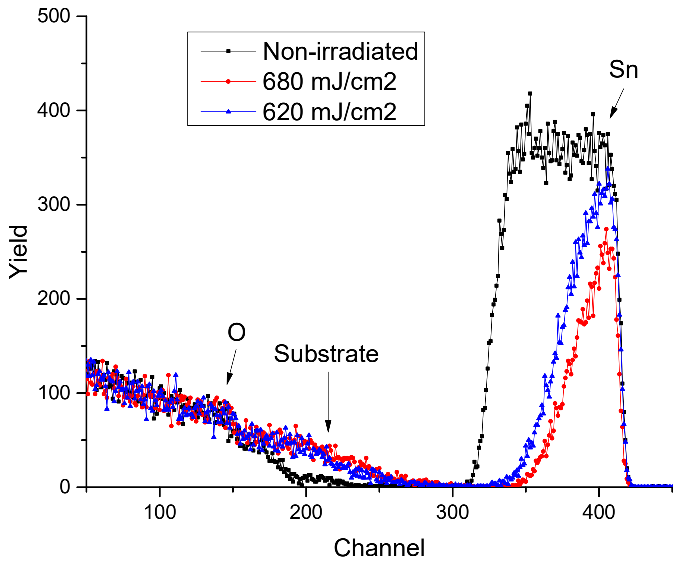

Figure S1. RBS spectra of FTO TEC-15 films on glass substrate

**Laser fluence (mJ/cm^2)**	**Total film thickness (10^{15} at/cm^2)**	**Film roughness (10^{15} at/cm^2)**
0	3000	500
620	1570	1450
630	1500	1450
636	1480	1450
642	1520	1480
650	1450	1450
655	1500	1500
660	1400	1500
667	1420	1410
680	1400	1400

Table S1. Total thickness and roughness of films measured by RBS.

2) Determination of the F-content by Nuclear Reaction Analysis (NRA)

To determine the F-content of the films, NRA measurements were carried out by using the $^{19}F(p, \alpha_0)^{16}O$ nuclear reaction with a proton energy of 1864 keV, beam diameter 1 mm and with a 300 mm^2 PIPS silicon detector placed at $\theta = 150^o$. The main advantage of this reaction is its high Q value, Q = 8114 keV, so the signal from the alpha particles appears at high energy, completely background free.

Absolute fluoride measurements by NRA can be compromised for several reasons. One is the possibility of fluoride loss during ion irradiation. Furthermore, it is difficult to find standards with well-known and long-term stable fluoride content. Finally, charge integration can be inaccurate in insulating samples. To avoid the last two problems, the measurements were taken without placing any filter in front of the detector so as to simultaneously obtain, in the same spectrum, the elastically scattered protons and the alpha particles from the nuclear reaction. As shown in Figure S2, the Sn and F (α_0) signals are completely resolved. Knowing that at this energy protons lose less than 20 keV in the SnO_2 films, we can consider the interaction cross-sections approximately constant along the entire path of the protons. In that case, if we compare the number of counts Σ of both signals, all charge integration effects cancel out and the atomic relative concentration F/Sn can be obtained from the formula:

$$\frac{[F]}{[Sn]} = \frac{\sum F}{\sum Sn}\frac{\sigma_{Sn}}{\sigma_F} \qquad \text{Equation (S1)}$$

Where σ_{Sn} is the Rutherford cross-section for Sn and σ_F is the cross section of the $^{19}F(p, \alpha_0)^{16}O$ nuclear reaction. For 1864 keV protons, σ_{Sn} = 1065 mb/sr while σ_F lies in the range 6.1-2.7 mb/sr [1-3]. For the calculations we have used the value of the more recent reference, σ_F = 4.2 mb/sr.

The main results of the NRA measurements are summarized in Table S2. From the F/Sn ratio, the absolute amount of F can be obtained by using the previous RBS results.

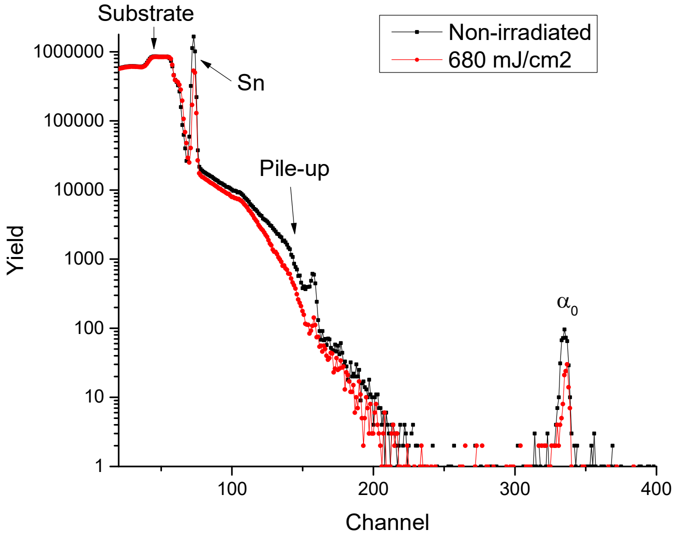

Figure S2. NRA spectra from non-irradiated and irradiated region with 680 mJ/cm^2.

Laser fluence (mJ/cm^2)	ΣSn (net) (x 10^6)	ΣF	[F]/[Sn] ±10%
0	4.12	473	0.029
620	1.88	196	0.026
630	1.72	157	0.023
636	1.68	162	0.024
642	1.69	164	0.025
650	1.65	171	0.026
655	1.56	173	0.028
660	1.42	157	0.028
667	1.35	137	0.026
680	1.29	124	0.024

Table S2. Elemental quantification estimates for all films derived from the NRA spectra

In comparison with the EDX results, the RBS+NRA measurements show an almost constant F-concentration for all analysed regions, regardless of the irradiated laser fluence. Moreover, the absolute concentration of F is also much lower than that obtained by EDX, with the ratio F/SnO_2 ~ 1%, which would suggest that a very large amount of F is desorbed from the samples during irradiation with the ion beam However, it is worth noting that the NRA spectra were obtained with a total integrated charge of 40 μC, in 2 consecutive steps of 20 μC each, and that, within the

statistical error, the number of counts in the α_0 peak remained constant in each measurements. Therefore, the potential losses of F would have had to occur during the first moments of irradiation, a fact that, although it cannot be ruled out, deserves a more in-depth study.

References

[1] D.Dieumegard, B. Maurel and G. Amsel, Nuclear Instrum.and Methods in Physics Res., Vol.168, p.93 (1980)

[2] V.Paneta, A.Kafkarkou, M.Kokkoris, A.Lagoyannis, Nucl. Instrum. Methods in Physics Res., Sect.B, Vol.288, p.53 (2012)

[3] I.Golicheff, Journal of Radioanalytical Chemistry, Vol.22, p.113 (1974)

3) XPS measurements on pristine and laser-processed samples

As indicated in Section 5 of the manuscript (Experimental methods), the composition of the near-surface region in pristine and laser-treated samples was also analyzed by X-ray photoelectron spectroscopy (XPS) in a SPECS spectrometer provided with a hemispherical analyser (DLSEGD-PhoibosHsa3500), using nonmonochromatic Mg Kα radiation line to excite the spectra in a normal configuration. Data were recorded with a 50 eV constant pass energy mode for the general survey spectra and 30 eV for high-resolution spectra. These spectra were calibrated in binding energy using the C1s photopeak associated with adventitious carbon surface contamination at 284.5 eV.

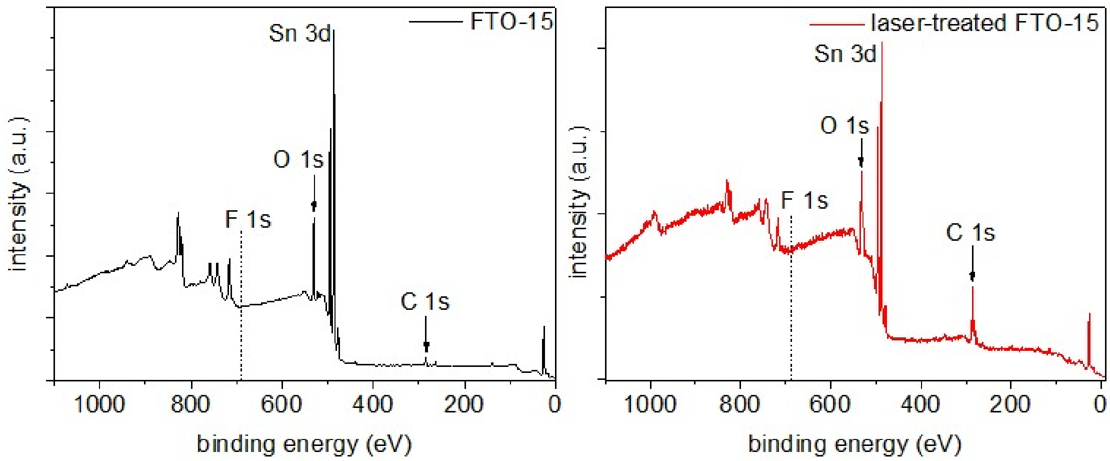

Figure S3. (left) XPS spectra of a pristine TEC-15 FTO sample and (right) a sample processed with with 420 fs at 650 mJ/cm^2.

Figure S3 (left) shows the spectrum corresponding to a pristine FTO TEC-15 sample where three distinct peaks corresponding to the C 1s (~285 eV), O 1s (~532 eV) and Sn 3d (~488 eV) binding energies can be identified, representing a carbon atomic concentration around 17% and a Sn/O ratio of 0.42, slightly lower than the stoichiometric expected value (~0.5), corresponding typically to a oxidated surface. The F 1s peak (~ 686 eV) is not observed. The measurement was repeated at several places of the pristine surface and, in no case, F was detected. Fig S3 (right) shows the equivalent survey spectrum performed in a region irradiated with 420 fs pulses at 650 mJ/cm^2, with an increase of the carbon contribution up to 46% and a Sn/O ratio of 0.19. This indicates that the laser treatment also promotes the adsorption of adventitious carbon and atmospheric organic species. The expected position of the F 1s contribution is indicated. No trace of F is observed in

this case neither in any of the 9 areas irradiated with fluences from 620 to 680 mJ/cm^2 that were also measured by NRA. We attribute the lack of F in the XPS measurements can be explained by the relatively low fluorine doping level typically present in FTO coatings and the limited surface sensitivity of XPS, i.e. the very shallow depth probed by XPS (a few nm). The near surface region appears to be strongly depleted in F due to its large mobility. We repeated these tests in samples exposed to Ar-ion bombardment at 5 kV for 15 and 30 min again achieving no signature of the presence of F, although carbon concentration decreased to the 1% and the Sn/O ratio reached a value of 0.63, consistent with a well-known oxygen depletion caused by a preferential sputtering of oxygen relative to tin. As we have already pointed out, ion bombardment seems capable to remove the F from the near-surface region samples at large depths (see FIB and NRA results) due to the lower mass and weaker bonding of fluorine doping species. Moreover, the laser irradiation process can induce local restructuring, desorption or redistribution of fluorine atoms at the surface, further reducing their detectable surface concentration.

4) Thermographic measurements at -10ºC in the climatic chamber for electrothermal de-icing performance tests

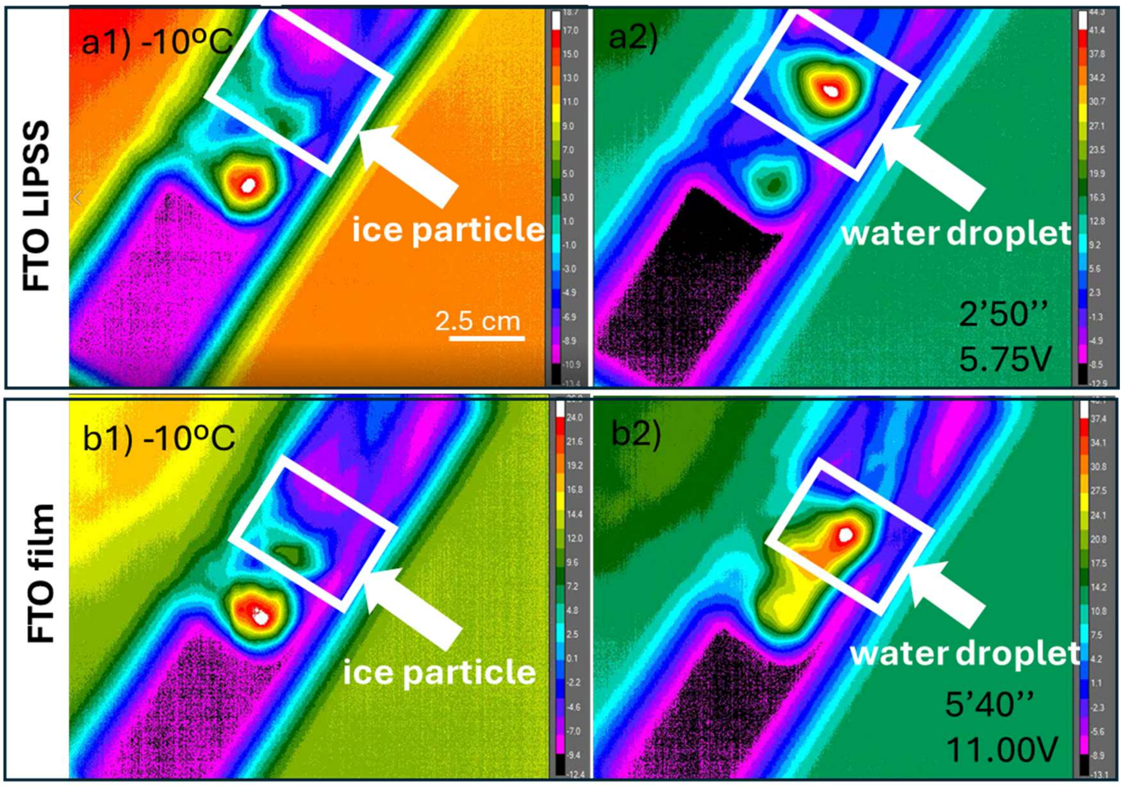

Figure S4. Thermographic maps of the FTO-structured LIPSS surface (a), and the pristine FTO film (b) before the de-icing experiment of a water droplet frozen onto both surfaces (a1, b1, respectively) and at the time when ice particle melting was evident (a2, b2, respectively). Samples temperature has been kept at -10 ºC. The white squares and arrows indicate the region where the complete device is located inside the climatic chamber, with the ice particle or water droplet on the surface, respectively, the location being observed with a temperature increase of more than 20 ºC.
